# Temperature Dependence of Solubility Predicted from Thermodynamic Data Measured at a Single Temperature: Application to α, β, and γ-Glycine

**DOI:** 10.1021/acs.cgd.1c01217

**Published:** 2022-02-08

**Authors:** Andrew Manson, Jan Sefcik, Leo Lue

**Affiliations:** † Department of Chemical and Process Engineering, University of Strathclyde, James Weir Building, 75 Montrose Street, Glasgow G1 1XJ, U.K.; ‡ EPSRC Continuous Manufacturing & Advanced Crystallisation (CMAC) Future Manufacturing Research Hub, University of Strathclyde, Glasgow G1 1RD, U.K.

## Abstract

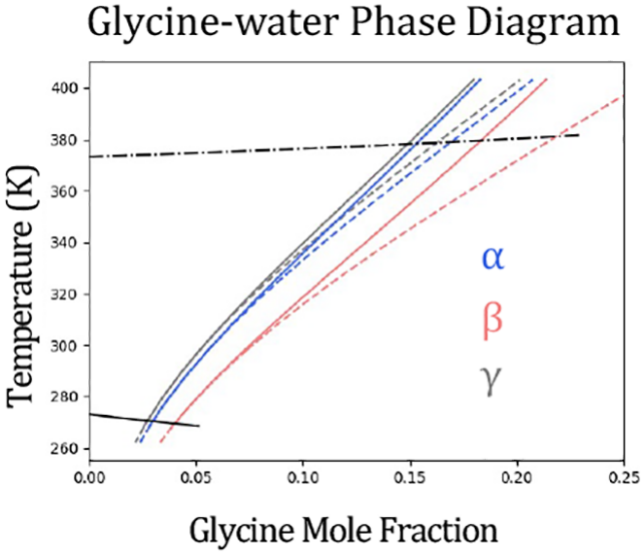

Understanding of solid–liquid equilibria for polymorphic systems is crucial for rational design and efficient operation of crystallization processes. In this work, we present a framework to determine the temperature dependent solubility based on experimentally accessible thermodynamic data measured at a single temperature. Using this approach, we investigate aqueous solubility of α, β, and γ-glycine, which, despite numerous studies, have considerable quantitative uncertainty, in particular for the most stable (γ) and the least stable (β) solid forms. We benchmark our framework on α-glycine giving predictions in excellent agreement with direct solubility measurements between 273–340 K, using only thermodynamic data measured at the reference temperature (298.15 K). We analyze the sensitivity of solubility predictions with respect to underlying measurement uncertainty, as well as the excess Gibbs free energy models used to derive required thermodynamic quantities before providing solubility predictions for β and γ-glycine between 273–310 and 273–330 K, respectively. Crucially, this approach to predict solubility as a function of temperature does not rely on measurement of solute melting properties which will be particularly useful for compounds that undergo thermal decomposition or polymorph transition prior to melting.

## Abstract 2

Understanding solid−liquid equilibria for polymorphic systems is crucial for the rational design and efficient operation of crystallization processes. A framework is presented to determine temperature dependent solubility based on experimentally accessible thermodynamic data measured at a single temperature. Using this approach, we investigate aqueous solubility of glycine polymorphs.

## Introduction

Glycine is the most simple amino acid^1,2^ known to crystallize in one of three polymorphs identified as γ, α, and β (in order of decreasing thermodynamic stability at ambient conditions^1,3^). Different crystallization techniques (i.e., cooling, evaporation, antisolvent, etc.) can be used to crystallize respective glycine polymorphs while additives (e.g., salts) and process conditions (e.g., stirring) can also be used to change the polymorphic outcome.^3^ Rational design and efficient operation of crystallization processes requires quantitative understanding of solid–liquid equilibria over a suitable range of operating conditions, as equilibrium solubility compositions and respective supersaturations need to be known for relevant solid forms.

The aqueous solubility of glycine polymorphs has been a topic of numerous investigations (see refs (2) and (4) for reviews). As pointed out in recent works,^3,5^ aqueous solubility data for α- and γ-glycine is largely inconsistent. Hence, direct measurements of the aqueous solubility of glycine polymorphs using conventional techniques appear challenging for the most stable solid form, γ-glycine, and even for α-glycine at elevated temperatures (above 330 K). This may be related to issues with mass transfer and particularly with very slow growth (and perhaps dissolution) rates at small supersaturations in highly concentrated solutions, so that it could be challenging to ensure that solid–liquid equilibrium is reached. For β-glycine, there is currently limited solubility data available, most likely due to rapid recrystallization to α-glycine.^6^ In turn, this then motivates our work investigating alternative approaches to prediction of solubilities using other thermodynamic data that may be accessible.

Measurements of thermodynamic properties other than solubility (e.g., enthalpy of solution, vapor pressure, etc.) exist over a fairly broad range of temperatures^2^ for the glycine–water system; however, the vast majority of measurements are reported around 298.15 K between dilute to moderate concentrations of glycine. Practical difficulties in working with glycine–water solutions at higher temperatures and moderate concentrations severely limit the availability of experimental data at elevated temperatures.

Thermodynamic models provide a means by which experimental measurements (e.g., solubility, heat capacity, etc.) can be used to make estimates beyond the specific conditions at which the measurements were performed and for properties other than those that were measured. In this approach, mathematical expressions are used to represent the free energy (e.g., Gibbs or Helmholtz free energy) of the system, and from this all thermodynamic properties of the system can be determined. Thermodynamic models are attractive, in theory, because they can greatly reduce the experimental effort required, for example, to determine the value of a specific physical property of a system over a broad range of conditions.

The theoretical basis and complexity of thermodynamic models can vary significantly, in part, depending on their application. At one extreme, there are fully empirical models with parameters that are adjusted to reproduce experimental data but which do not have any direct physical interpretation. For example, when solubility is assumed to be a polynomial function of temperature and the coefficients are regressed against a series of direct solubility measurements at different temperatures.^7^ The resulting model is used to predict solubility at conditions not covered by experiment through interpolation and extrapolation.^8,9^ While this approach can be quite useful in practical applications, its reliability outside the range of conditions covered by the experimental data is uncertain.

At the other extreme is the use of free energy models that are firmly rooted in molecular statistical mechanics with physical meaningful parameters that are related to properties such as molecular sizes or interactions strengths. This allows the a priori estimate of the values of the parameters, or at least provide physical bounds as to their possible range, without need for experimental input. While these more theoretically based approaches are expected to have a greater range over which the results can be reliably extrapolated, there is almost always a need to adjust the model parameters, due to approximations and simplifications made during their derivation, in order to quantitatively reproduce experimentally measured properties.

Within this approach, for the prediction of solubility, a thermodynamic model is required for the liquid phase, and a separate model is required for the solid phase. For glycine–water mixtures, the Pitzer,^10^ mean spherical approximation (MSA),^10^ modified Wilson,^11^ and PC-SAFT,^12,13^ as well as many other, models have been used successfully to describe the liquid phase. Typically, the parameters of these models need to be regressed across a large collection of thermodynamic measurements over a wide range of conditions. The predictions of these models can then depend sensitively on the precise forms chosen for these parameters, such as how they depend on temperature and composition. What is needed is a manner to assess how far an experimental measurement can be reliably extrapolated.

In addition, a thermodynamic model is required for the solid, which typically rely on pure solute melting enthalpy, melting temperature and heat capacity (solid and liquid solution reference state, typically taken as the pure liquid solute).^8,14^ However, this approach is limited for systems where melting properties are not experimentally accessible using conventional approaches, due to thermal decomposition (as is the case for glycine), polymorph transition, etc. However, it should be noted that recently, novel approaches have been reported recently that mitigate these issues.^13^ Regardless, simplifications employed during model derivation can lead to significant errors in solubility predictions at temperatures far from the solute melting temperature.

In this work, we develop a novel approach to determine the temperature dependence of solubility, which, in principle, relies entirely on thermodynamic data collected at a single temperature by approximation of parameter dependencies through first-order Taylor series expansions. This is advantageous in the sense that it limits the amount of thermodynamic data required to make solubility predictions. With this approach, we provide an estimate for the aqueous solubility of α-glycine, from its eutectic point to the solid-solution–vapor triple point (i.e., intersection of solubility and boiling curve). This is based on enforcing consistency with thermodynamic measurements for a broad range of properties at 298.15 K. For β and γ-glycine, where solubility measurements are inconsistent or limited, we develop methods for estimating the solubility at 298.15 K, as well as its dependence on temperature, using ancillary thermodynamic data.

The remainder of the Article is structured as follows: First, the relevant thermodynamic theory is introduced. Then, the relevant approximation is described and an interpretation of model parameters is provided in the context of experimental measurements. Then, a review of thermodynamic data (including solubility, activity, enthalpy, and heat capacity) and results from excess free energy model fitting are given, followed by solubility predictions for α-glycine. The sensitivity of our approach to uncertainty in experimental data and excess free energy model choice is then discussed. Finally, the extension of the method to β- and γ-glycine is presented.

## Theory

We consider a solute (denoted as 1), which is dissolved in a solvent (denoted as 2). At sufficiently high concentrations, the solute precipitates as a pure solid. The temperature dependence of the mole fraction of the solute *x*_1,sat_(*T*) at saturation can be expressed by the following relation:
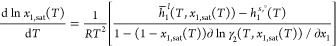
1
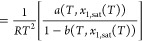
2where *R* is the gas constant, *h̅*_1_^*l*^ (*T*, *x*_1,sat_(*T*)) is the partial molar enthalpy of the solute in a saturated solution at temperature *T* and *h*_1_^*s*,°^(*T*) is the molar enthalpy of the pure solid at *T* (which is not a function of solution composition). The solute activity coefficient γ_1_ is defined with the chemical potential of the solute written as *βμ*_1_^*l*^ = *βμ*_1_^°,*l*^(*T*) + ln *x*_1_γ_1_, where μ_1_^°,*l*^(*T*) is the chemical potential of pure liquid solute at temperature *T*. The quantity *h̅*_1_^*l*^(*T*, *x*_1,sat_(*T*)) – *h*_1_^*s*,°^(*T*) is sometimes referred to as the “differential heat of solution”, and the quantity (1 – *x*_1,sat_(*T*))∂ ln γ_2_(*T*, *x*_1,sat_(*T*))/∂*x*_1_ is related to the variation of the solvent activity coefficient (defined through the relation *βμ*_2_^*l*^ = *βμ*_2_^°,*l*^(*T*) + ln *x*_2_γ_2_, where μ_2_^°,*l*^(*T*) is the chemical potential of pure solvent at temperature *T*) with composition, evaluated at saturation temperature and composition. For convenience, we refer to *h̅*_1_^*l*^(*T*, *x*_1,sat_(*T*)) – *h*_1_^*s*,°^(*T*) as *a*, and (1 – *x*_1,sat_(*T*))∂ ln γ_2_(*T*, *x*_1,sat_(*T*))/∂*x*_1_ as *b*. In principle, both *a* and *b* are experimentally accessible, and if they were known in addition to a single solubility point, the solubility at any temperature could be predicted.

Expressions equivalent to eq 1 have been reported by various authors;^15−18^ however, it can be derived by considering that, at thermodynamic equilibrium, the chemical potential of a pure solid solute is equal to the chemical potential of the solute in a saturated solution (at the same temperature):
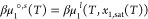
3where β = (*RT*)^−1^, *R* is the gas constant, μ_1_^°,*s*^(*T*) is the chemical potential of pure solid solute and μ_1_^*l*^(*T*, *x*_1,sat_ (*T*)) is the chemical potential of the solute in a saturated solution. Equation 3 is valid at any point along the solubility curve. More generally, small changes in the chemical potential terms can be expressed as
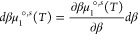
4
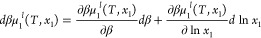
5which results from the fact that they are continuous functions of *T* and *x*_1_. Combining eqs 4 and 5, while noting that eq 3 can be expressed as *dβμ*_1_^°,*s*^(*T*) = *dβμ*_1_^°,*l*^(*T*, *x*_1,sat_(*T*)), gives
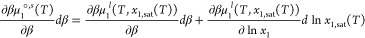
6which, again, is valid along the solubility curve. Going further
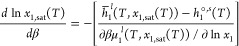
7where we make use of the Gibbs–Helmholtz relation *h̅*_*i*_ = ∂*βμ*_*i*_/∂β, where *h̅* is the partial molar enthalpy of species *i*. Focusing on ∂*βμ*_1_^*l*^(*T*, *x*_1,sat_(*T*))/∂ ln *x*_1_ gives
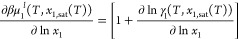
8which, coupled with eq 7,
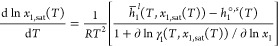
9which is an expression identical with that reported previously.^15−18^ Since data relating to the activity of solvent in solution is more widely reported, we use the Gibbs–Duhem relationship to replace the solute activity coefficient term:
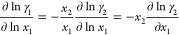
10noting that we make the arbitrary choice to use the partial derivative with respect to *x*_1_, rather than ln *x*_1_, which when combined with eq 9 gives eq 1. For interested readers, we report another derivation in our Supporting Information, based entirely on earlier work found in ref (17). As an aside, eq 1 is sometimes reported in the form:
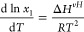
11where *ΔH*^vH^ is referred to as the “van’t Hoff enthalpy of solution”.^18,19^

The utility of eq 1 is 2-fold. First, if the solubility of a compound is known as a function of temperature, the right side can be determined, which directly leads to an estimate of the differential heat of solution and related solution properties.^17,20^ Alternatively, if the right side were known as a function of temperature and solution composition, the solubility as a function of temperature could be calculated by integration of the equation, given the solubility at one temperature as a starting point.^21^

In general, *a* and *b* in eq 2 are functions of both temperature and composition; however, it is unclear what those dependencies are. Ideally, we could collate sufficient experimental data to develop functional expressions for both parameters (i.e., across the entire range of temperature and solubility); however, in practice this would require significant effort. In addition, it is unclear if techniques are available that allow evaluation of the required measurements at extremes of temperature and solution composition.

In the absence of sufficient data to correlate functions for model parameters, we can approximate their temperature and composition dependence using Taylor’s theorem, which is a mathematical technique used to locally approximate analytic, multivariate functions in terms of their partial derivatives evaluated at a chosen reference point.

For convenience, we apply the first-order approximation of Taylor’s theorem, which, for a two-variable continuous function, is given as
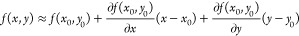
12
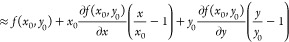
13

It should be noted that the first-order expansion will limit the accuracy of approximation far from the chosen reference point. For the purpose of this work, we perform the following expansions for *a* and *b*, respectively:
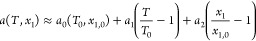
14
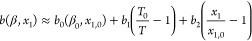
15where all coefficients are defined in the Supporting Information.

We have chosen to approximate *a* (*T*, *x*_1_) in terms of *T* and *x*_1_, since ∂*h*/∂*T* = *c*_*p*_, which makes it possible to use heat capacity data to evaluate *a*_1_, while we have chosen to approximate *b*(β, *x*) in terms of β and *x* because it allows us to relate *a*_2_ and *b*_1_. However, it should also be noted that the expansion can be performed in many other ways.

Substituting eqs 14 and 15 into eq 2, the solubility relation becomes
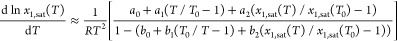
16from which the solubility at any temperature can be predicted by numerical integration if all model coefficients and the solubility at *T*_0_ is known.

For notational convenience, the composition dependence of coefficients in eq 16 will be represented by *x*_1_ and *x*_1,0_ in place of *x*_1,sat_ (*T*) and *x*_1,sat_ (*T*_0_), respectively, throughout the remainder of the paper. However, it should be emphasized that these refer to the quantities evaluated at or along the solubility curve.

### Relationship Between Model Coefficients and Measured Data

It is possible to relate each of the coefficients in eq 1 to various experimentally measurable thermodynamic quantities. Depending on the coefficient, the required thermodynamic quantities may be accessible through a direct measurement (e.g., enthalpy of solution to infinite dilution) or by regressing an excess Gibbs free energy model to different types of measurements and using the model to derive necessary quantities (e.g., using vapor pressure data to evaluate ∂ ln γ_2_(*x*_1_)/∂*x*_1_) It should be noted that there are many ways in which experimental data could be used to evaluate the coefficients in eq 16, resulting from the many relationships between thermodynamic quantities. However, for the purpose of this work, we relate coefficients to experimental measurements performed at a single temperature. For convenience, all *a* coefficients and all *b* coefficients are discussed together.

#### Determination of *a* Coefficients

The *a* coefficient can be expanded as
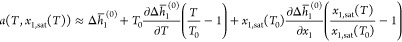
17where *Δh̅*_1_^(0)^ = *h̅*_1_^*l*^(*T*_0_, *x*_1,sat_(*T*_0_)) – *h*_1_^*s*,°^(*T*_0_) could be evaluated directly if “differential heat of solution” measurements were available; however, in the absence of these measurements, it can be shown that
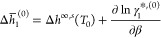
18where *Δh*^∞,*s*^ is the enthalpy of solution when forming an “infinitely dilute” solution.^22^ It should be noted that we introduce γ_1_^*^ = γ_1_/γ_1_^∞^ as the solute activity coefficient defined relative to an infinitely dilute solution, where γ_1_^∞^ = lim_*x*_1_ →0_γ_1_. The quantity ∂ ln γ_1_^*,(0)^/∂β = ∂ ln γ_1_^*^(*T*_0_, *x*_1,sat_ (*T*_0_))/∂β can be evaluated by regressing an excess Gibbs free energy model to “enthalpy of dilution” data (for cases where the enthalpy of dilution is given per mole of solute in solution) via
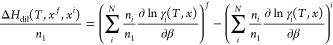
19In addition, since *Δh*^∞,*s*^(*T*_0_) is independent of composition, it can be shown that
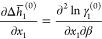
20which can be evaluated from the same excess Gibbs free energy model regressed to enthalpy of dilution data. Given that *c*_p_ = ∂*h*/∂*T*, it can be shown
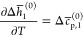
21where *Δc̅*_*p*,1_^(0)^ = *c̅*_*p*,1_^*l*^(*T*_0_, *x*_1,sat_(*T*_0_)) – *c*_*p*,1_^*s*,°^(*T*_0_), *c*_*p*,1_^°,*s*^ is the molar heat capacity of crystalline glycine, which can be measured directly, and *c̅*_*p*,1_^l^ is the partial molar heat capacity of glycine in solution which can be interpreted as
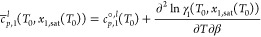
22which, given an excess Gibbs free energy model, can be estimated by regressing solution heat capacity data, since
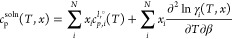
23

#### Determination of *b* Coefficients

In the context of this work:
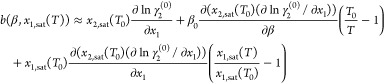
24where again ln γ_2_^(0)^ = ln γ_2_(*T*_0_, *x*_1,sat_(*T*_0_)) is introduced for notational convenience. It can be shown that
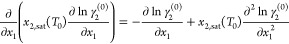
25which can be evaluated by regressing an excess Gibbs free energy model to “activity” related measurements (i.e., vapor pressure, isopiestic molality, etc.) taken at the reference temperature and at various compositions, and
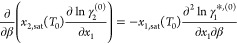
26which can be evaluated in the same way as eq 20.

### Excess Gibbs Free Energy Models

Several parameters required to estimate the coefficients in eq 16 are derivatives of continuous thermodynamic quantities. Given the discrete nature of many experiments, estimation of required parameters requires correlation of data using models. In the context of thermodynamics, the excess Gibbs free energy is a concept that each of the required quantities can be related to. Historically, many attempts have been made to derive physically interpretable excess Gibbs free energy models and as such, for the purpose of this work, we use the Scatchard–Hildebrand and Scatchard–Hildebrand–Flory–Huggins excess Gibbs free energy models.^23^

#### Scatchard–Hildebrand

The Scatchard–Hildebrand excess free energy model (for a binary solution comprising glycine and water) is given by
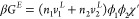
27where *v*_*i*_^*L*^ is the molar volume of component *i*, ϕ_*i*_ is the volume fraction of component *i*, and χ′ is a binary interaction parameter. This gives
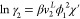
28
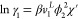
29For convenience, we define χ(β) = χ′*βv*_2_^*L*^ and v* = *v*_1_^*L*^/*v*_2_^*L*^, which gives
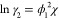
30
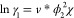
31where we have assumed χ to be a function of temperature, while the molar volumes (i.e., v*) are independent of temperature and composition. These assumptions make it straightforward to use the same model throughout the correlation of different thermodynamic data. In addition
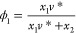
32
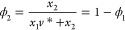
33The excess enthalpy of this model, assuming χ′ is a function of temperature only and v* is independent of temperature and composition, is given by
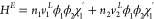
34and the excess heat capacity is given by
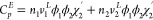
35where χ′_1_ = ∂χ′/∂β, and χ′_2_ = ∂^2^χ/∂*T*∂β.

#### Scatchard–Hildebrand–Flory–Huggins

To account for differences in molecular size we can add a “Flory–Huggins” term to the Scatchard–Hildebrand free energy expression; which, for a binary solution, is given by
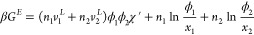
36which gives
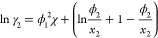
37
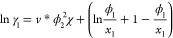
38It should be noted that *H*^*E*^ and *C*_*p*_^*E*^ are the same for both the Scatchard–Hildebrand–Flory–Huggins and Scatchard–Hildebrand when χ′ is a function of temperature only and v* is a constant independent of temperature and composition, which is an assumption used in this work.

## Results and Discussion

### Solubility Review

There are numerous reports of glycine–water solubility measurements in the literature. Some articles present data for an identified polymorph (see ref (24) for α, β and γ, refs (25−29) for α and γ, refs (2,30,31) for α and ref (32) for γ), while others refer only to “glycine” (see refs (9 and 33−39)), which is problematic, since crystal polymorphs have different solubility in the same solvent at the same temperature and pressure.

Some of this data has been reviewed recently;^2,4^ however, both reviews have specific areas of focus and limitations. Datta et al.^4^ make no distinction between glycine polymorphs, choosing to review only data that has been identified as “glycine”. Their review does not include any data labeled with glycine polymorphs, excluding a number of data sets from their review.

Rowland^2^ reviewed solubility data as part of their work on an equation of state model parameters, as well as the standard state properties for the glycine–water solution. Rowland’s review is restricted to data assumed to be α-glycine, which includes data points reported as α-glycine, as well as data where the glycine polymorph is unspecified; however, β and γ-glycine were out of the scope of the work.

**Figure 1:**
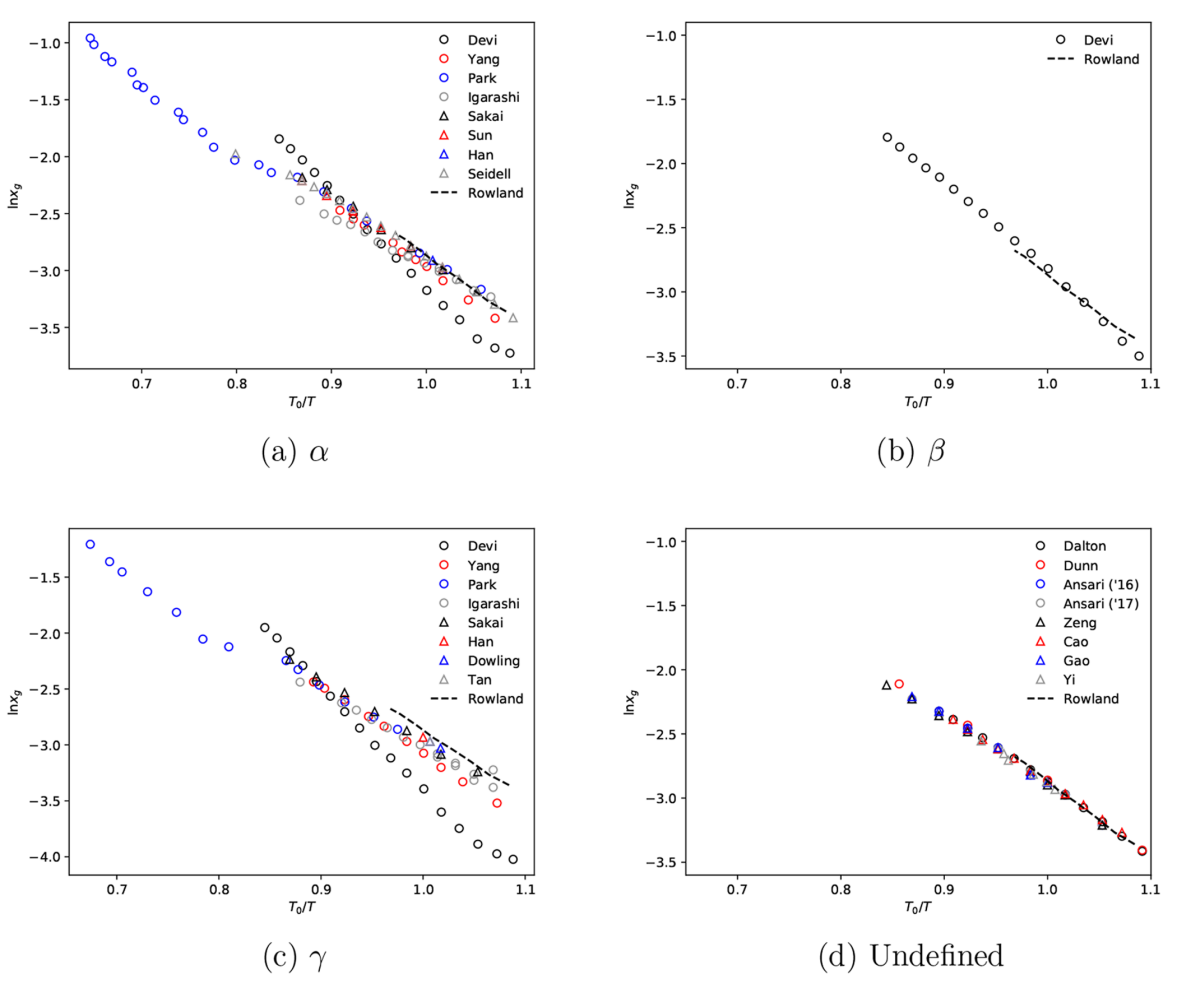
“Van’t Hoff” style plot of literature solubility data^9,24−39^ for α-, β-, γ-, and undefined glycine between 270–430 K. Note that *T*_0_ = 298.15 K. The black dashed line is the α-glycine solubility estimate from ref (2) and is shown in all plots as a guide.

The primary aim of our glycine–water solubility review is to consider other glycine polymorphs alongside α-glycine, as well as any missing data not included previously, especially at elevated temperatures where glycine solubility data are sparse. We proceeded as follows: first, if the solubility data had the polymorph labeled (e.g., α, β, or γ) it was included. Then, if the solubility data was for an undefined glycine polymorph, it was included only if the data set included data above 330 K. Data meeting either of these criteria are presented in Figure 1.

We note that some solubility compilations (e.g., see Table A.5 in ref (7)) show glycine solubility up to 373 K, without providing a source reference. However, we believe data in ref is based on an extrapolation of lower temperature data as reported early on;^33^ this was also before polymorphism of glycine was established.

From Figure 1, each data set shows the solubility of glycine in water increasing with temperature as generally expected. For data labeled as either α or γ-glycine, there are a similar number of data sets because of various studies reporting measurements for both polymorphs together. In addition, it can be seen that data is sparse above 350 K, with only one data set for α and γ-glycine, respectively. Interestingly, the labeled polymorph data sets have greater variability, while the undefined polymorph data sets appear more consistent. It is unclear why this is the case; however, following our own assessment and in agreement with Rowland, we attribute the undefined data sets as α-glycine. On the basis of the slope of their data, we observe that data from Devi and Igarashi for both α and γ-glycine is inconsistent with each other and other data reported in the literature.

Based on our review, there is only one report of β-glycine in the literature; however, it is interesting to note reports that direct measurement of β-glycine in pure water are challenging due to kinetic instability in water and resulting rapid transformation to α-glycine.^6,40^ Taking Rowland’s estimate for α-glycine as a guide, it appears data reported as β-glycine could be α-glycine.

### Thermodynamic Properties

As discussed above, Rowland^2^ published a comprehensive review of thermodynamic data for glycine–water mixtures. Much of the data presented there corresponds to data required to evaluate the coefficients in eq 16 and as such, the work was used as a reference for thermodynamic data. An initial review indicated that most of the thermodynamic data for glycine–water solutions has been collected at 298.15 K, as such we chose *T*_0_ = 298.15 K as the start point for our Taylor expansion. Following a further review of literature, any additional data required to evaluate the coefficients in eq 16 was found elsewhere.

Solubility at the chosen reference temperature is a crucial thermodynamic property required to make predictions with the approach presented in the theory section. For α-glycine, we have chosen to take the value reported by Rowland^2^ (i.e., 3.324 mol kg^–1^ of water or 0.056 (glycine mole fraction) at 298.15 K), derived from a thermodynamically consistent, semiempirical fit to a broad range of experimental data (including solubility), which we believe to be the best estimate available in literature.

It should be noted that, for the purpose of this work, we model solution properties separately (i.e., ln γ_w_ is modeled independently of *ΔH*^dil^/*n* and *c*_p,soln_), rather than developing a model to simultaneously describe all thermodynamic data. Development of a comprehensive model is out with the scope of this work, and the approach described here is computationally convenient and ensured experimental data was modeled accurately.

In the following section, thermodynamic data used to evaluate the coefficients in eq 16 is described. This includes measurements from which the solvent activity coefficient can be derived, enthalpy of dilution, and solution heat capacity, as well as measurements for enthalpy of solution to infinite dilution and crystalline glycine polymorph heat capacity (both at 298.15 K).

#### Activity Coefficient Data, Fits, and Derived Properties

**Figure 2:**
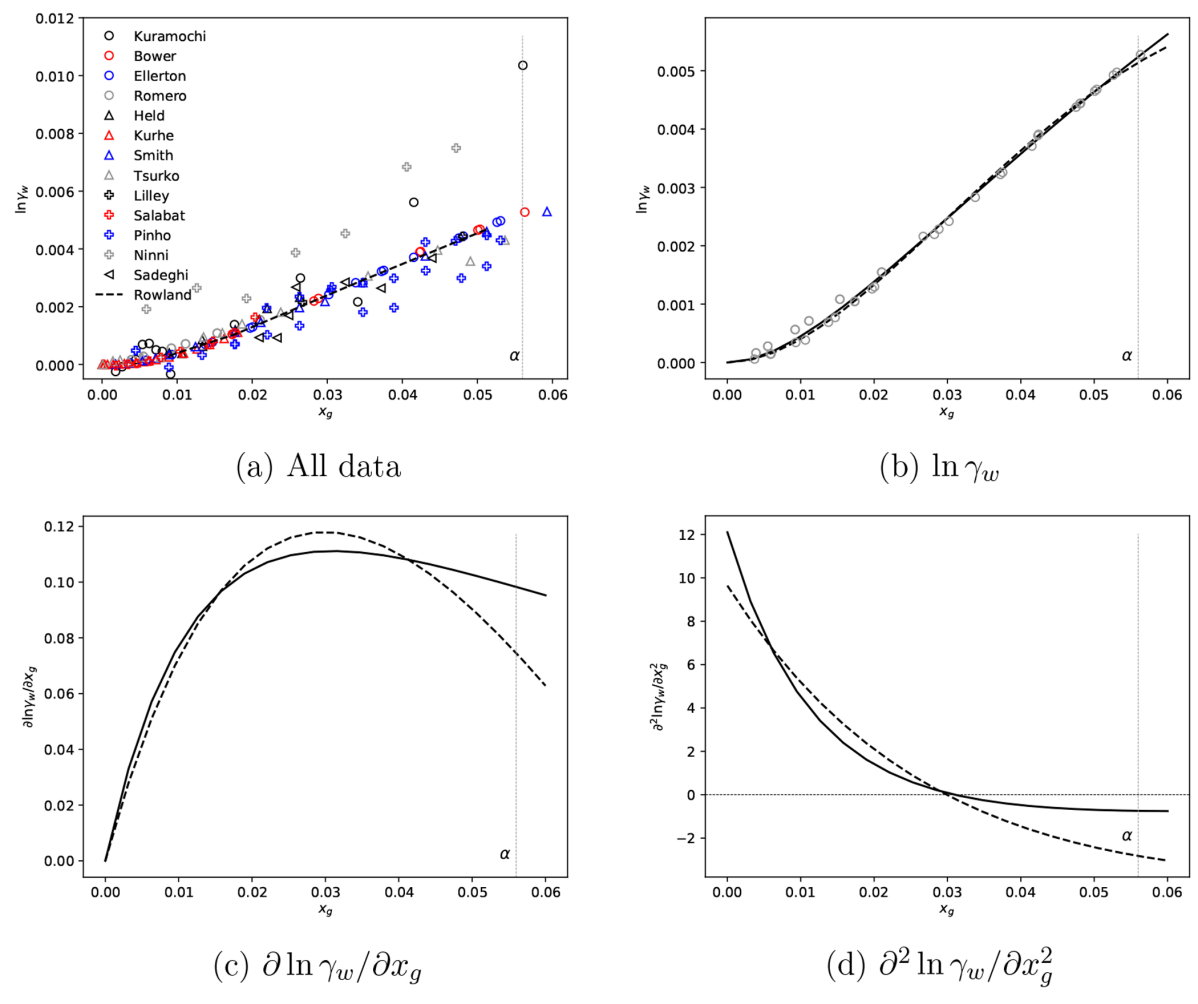
(a) Literature glycine–water solution activity data at 298.15 K,^10,12,41−50^ (b) model fits to selected data activity data, (c) 1st-order partial derivative for fitted models with respect to concentration, and (d) 2nd-order partial derivative for fitted models with respect to concentration. Solid lines are the predictions of the Scatchard–Hildebrand model, dashed lines are the predictions of the Scatchard–Hildebrand–Flory–Huggins model, and symbols are converted experimental data from the literature.

From the review published by Rowland, 12 data sets^10,12,41−50^ were found from which the water activity coefficient as a function of solution composition can be derived. For seven of the data sets, the water activity coefficient γ_w_ was derived from isopiestic or osmotic vapor pressure measurements and subsequent estimation of the osmotic coefficient in glycine–water solutions of various compositions at 298.15 K using
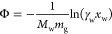
39where *x*_w_ is the mole fraction of water, *M*_w_ = 0.018 kg mol^–1^ is the molecular weight of water, and *m*_g_ is glycine molality. Four data sets reported values for water activity *a*_w_ at various glycine–water solution concentrations (at 298.15 K), which were converted to activity coefficients via
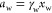
40The final data set reported static vapor pressure measurements for glycine–water solutions of varying composition at 298.15 K. The water activity coefficient was determined from the modified Raoult’s law (assuming the vapor was ideal and comprised only of water)
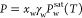
41where *P* is the pressure and *P*_w_^sat^(*T*) is the saturated vapor pressure of pure water at 298.15 K, taken as the value provided in the data set. All data, converted to ln γ_w_ and plotted as a function of solution composition, are given in Figure 2a.

From Figure 2a, the general trend indicates that ln γ_w_ increases with glycine mole fraction, meaning that γ_w_ > 1. Excluding the data from Ninni, the data appears to follow the same trend; however, there is significant variability between datasets, particularly above *x*_g_ = 0.03. In general, evaluations made from isopiestic vapor pressure measurements appear to be more consistent in terms of internal scatter. A review of data included in Rowland’s global fit indicates that all data used was measured using an isopiestic vapor pressure technique, except from the single point that was measured using vapor pressure osmometry. For the purpose of this work, we assume the data chosen by Rowland to be the most reliable and use this to regress the Scatchard–Hildebrand and Scatchard–Hildebrand–Flory–Huggins models, respectively.

From Figure 2b, both the Scatchard–Hildebrand and Scatchard–Hildebrand–Flory–Huggins models appear to fit the activity coefficient data well. However, the choice of model has a significant impact on behavior of the first and second partial derivatives and their values at glycine polymorph solubility. For example, the first partial derivative evaluated at the estimated α-glycine solubility is 0.098 and 0.075 for the Scatchard–Hildebrand and Scatchard–Hildebrand–Flory–Huggins model, respectively, while the second partial derivative is −0.74 and −2.82. Given that both partial derivatives are parameters used to evaluate solubility relation coefficients, the differences suggest that, in the case of discrete data, the choice of model can impact the predictive ability.

#### Enthalpy of Dilution Data, Fits and Derived Properties

**Figure 3:**
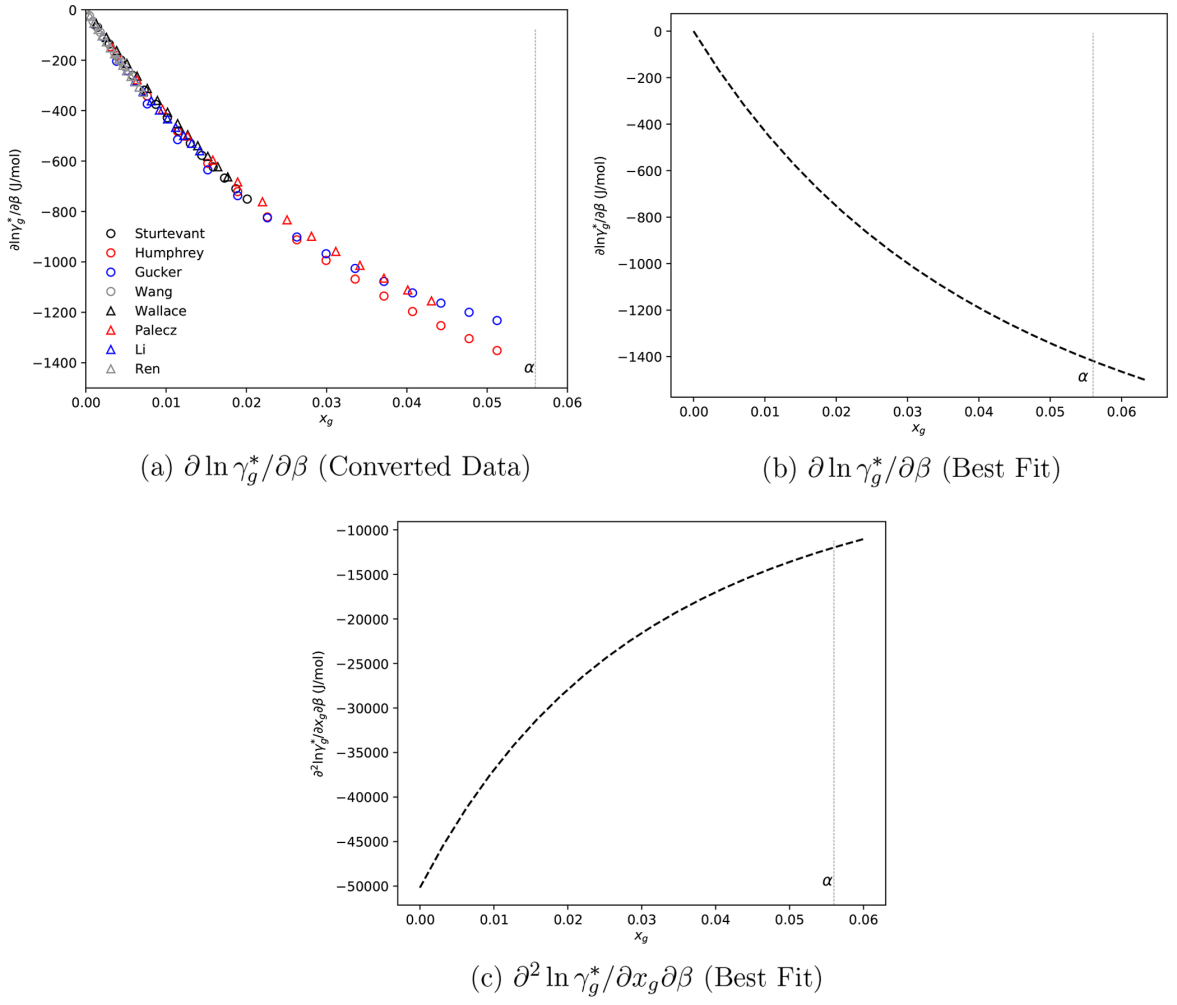
(a) Literature glycine–water solution enthalpy of dilution processed data at 298.15 K and (b, c) ∂ ln γ_g_^*^ / ∂β and ∂^2^ ln γ_g_^*^/∂*x*_*g*_∂β derived from Scatchard–Hildebrand best fit to data in panel a. Open symbols are values derived from fits to literature data,^51−58^ and gray dashed vertical lines indicate α-glycine solubility at 298.15 K.

Again, following Rowland there are at least 8 data sets^51−58^ available in the literature reporting enthalpy of dilution measurements. For the purpose of review, each data set was correlated by eq 19 to allow direct comparison, noting that raw measurements cannot be compared graphically. From the best fit to each data set, points corresponding to ∂ ln γ_g_^*^/∂β were generated corresponding to the range over which measurements were reported. The resulting processed data is presented in Figure 3a.

From Figure 3a, data generated for best fits to each data set appear consistent from 0–0.02 mole fraction. However, beyond 0.02, there is uncertainty resulting from a lack of data and inconsistency between the data that are available. This is problematic because an estimate the partial derivative of this curve at the estimated solubility is required to evaluate model parameters, and thus, extrapolation is necessary, which may introduce uncertainty.

Again, eq 19 was regressed against the aggregated data set (i.e., the data sets used by Rowland), and the results are presented in Figure 3b, with ∂^2^ ln γ_g_^*^/∂*x*_g_∂β in Figure 3c. It should be noted that our best fit estimate for ∂ ln γ_g_^*^/∂β is in excellent agreement with that presented in ref (52).

#### Solution Heat Capacity Data, Fits and Derived Properties

**Figure 4:**
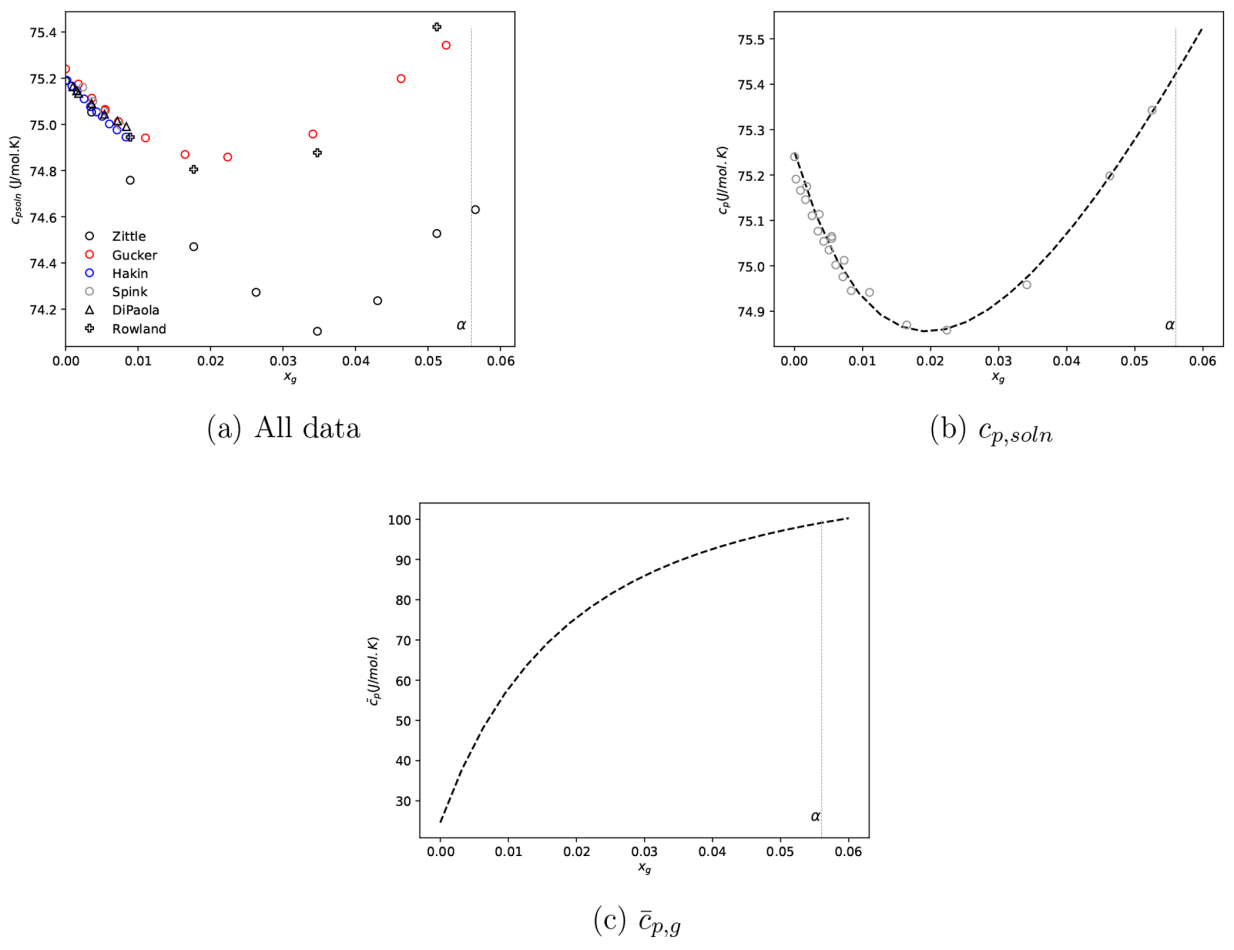
(a) Glycine–water solution heat capacity data at 298.15 K^20,59−62^ and (b, c) c_p_^soln^ and *c̅*_p,g_ derived form Scatchard–Hildebrand best fit to selected data in panel a. Gray dashed vertical lines indicate α-glycine aqueous solubility at 298.15 K.

Rowland references 9 data sets reporting measurements relating to the heat capacity of glycine–water solutions. However, only 5^20,59−62^ have data for solutions at 298.15 K. These data are presented in Figure 4a. It should be noted that, where data was reported in terms of the “apparent molar heat capacity”, data has been converted to solution heat capacity using pure water molar heat capacity of 75.2 J mol^–1^.

From Figure 4a, 4 data sets appear consistent between 0 and 0.01, corresponding to the data used by Rowland in their global fit. For the purpose of this work, we use these data sets. It should be highlighted again that data does not extend to solution compositions required to accurately estimate properties at estimated solubility. In addition, there is only one data set that extends beyond a solution composition of 0.01. Thus, we have to extrapolate regressed models to evaluate required parameters The fit and corresponding partial molar heat capacity are given in Figure 4b and 4c.

#### Enthalpy of Solution and Pure Crystal Heat Capacity

Direct measurements of the enthalpy of solution (to infinite dilution) and pure solid molar heat capacity at 298.15 K, are required to evaluate the coefficients in eq 16. Both have been reported in the literature and are presented in Table 1. We note that both *Δh*^∞,*s*^ and *c*_p,298.15_ has been measured for α-, β-, and γ-glycine, respectively. However, we also note that each property has been measured only once and not validated by additional measurements.

**Table 1:** Enthalpy of Solution (to Infinite Dilution) and Crystal Molar Heat Capacity Data for α-, β-, and γ-Glycine at 298.15 K

polymorph	*Δh*^∞,*s*^ (J mol^–1^)^22^	*c*_*p*, 298.15_ (J/mol.K)^63^
α	14523 ± 76	99.23
β	14198 ± 73	98.69
γ	14791 ± 84	96.00

### α-Glycine Solubility Predictions

**Figure 5:**
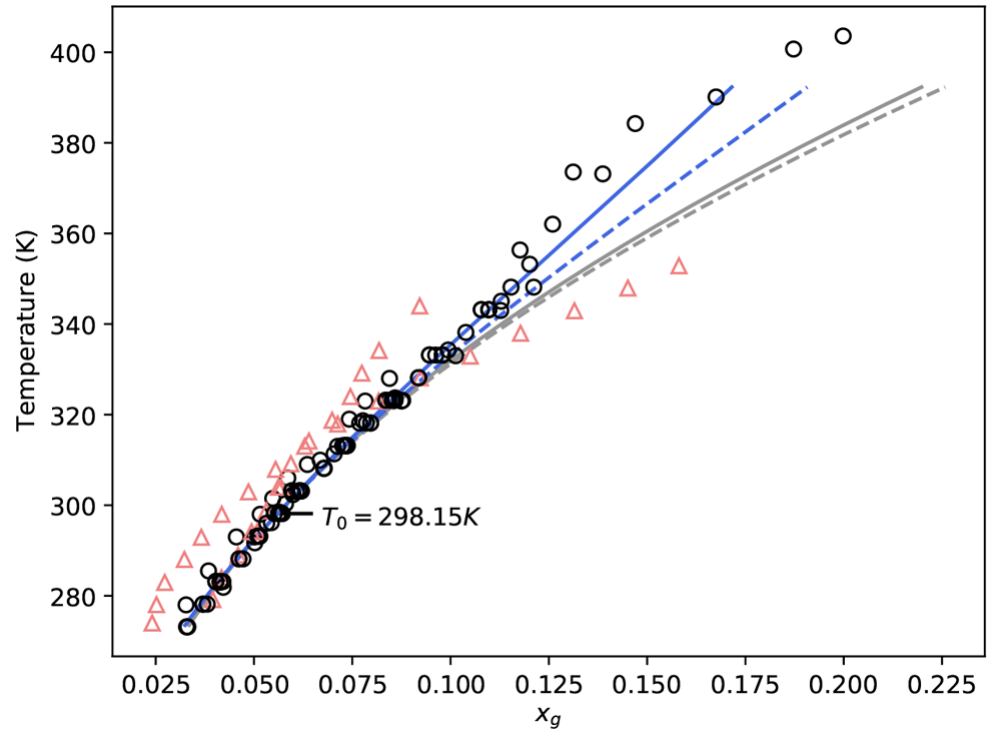
Temperature-dependent α-glycine solubility predictions for various data modeling approaches. (Gray lines: 0th-order predictions. Blue lines: 1st-order predictions. Dashed lines: Scatchard–Hildebrand predictions. Solid lines: Scatchard–Hildebrand–Flory–Huggins predictions. Black open symbols: Direct measurements labeled as α-glycine^25,26,28−30^ and undefined glycine.^9,33−39^ Refs (24) and (27) highlighted with red triangles due to being inconsistent with rest of available data.)

The solubility of α-glycine was predicted as a function of temperature by numerically integrating eq 16 with the coefficient values presented in the Supporting Information, and predictions are shown in Figure 5. It should be noted that the solubility was also estimated with *a*_0_ and *b*_0_ only, which is referred to as the “0th-order” prediction, while predictions based on eq 16 are referred to as “1st-order” predictions.

From Figure 5, the zeroth and first-order approaches both predict an increasing solubility with temperature. From 280–330 K, irrespective of the excess Gibbs free energy model used to estimate model parameters, both zeroth and first-order approaches show good agreement in terms of solubility; however, beyond this, predictions diverge. Although solubility data is limited above 340 K, the first-order corrections shift solubility predictions toward available measurements, indicating that the first-order corrections improve those made by the zeroth-order model.

We note that our solubility predictions are consistent with the majority of available direct measurements, as shown in Figure 5. However, our predictions vary considerably compared to refs (24) and (27), shown as red open triangles. This supports initial observations detailed in our solubility review, where the slope of both data sets was inconsistent with other literature data when plotted on a van’t Hoff plot. As such, both data sets will be omitted from plots in the remainder of this work.

The effect of the excess Gibbs free energy model used to evaluate the coefficients in eq 16 is shown in Figure 5. For the zeroth-order predictions, model impact is limited—becoming significant around 380 K. However, the impact of model selection is greater for the first-order predictions. The corrected models begin to diverge at approximately 340 K. At 400 K, the solubility is predicted as 0.20 and 0.18 (glycine mole fraction) for the Scatchard–Hildebrand and Scatchard–Hildebrand–Flory–Huggins models, respectively.

Differences between the chosen excess Gibbs free energy models used to derive thermodynamic quantities is specific to quantities derived from fits to solvent activity coefficient data. Previously, it was shown that, although both models (Scatchard–Hildebrand and Scatchard–Hildebrand–Flory–Huggins) appeared to fit water activity coefficient data well, the first and second partial derivatives (with respect to glycine mole fraction) were significantly different. It is interesting to note that, despite this, both models produce consistent predictions near 298.15 K.

On the basis of the available solution thermodynamic data and solubility, we believe further progress could be made on assessing the performance of the approach detailed above in two ways. First, with more accurate high temperature solubility data, since most solubility data above 340 K comes from the same source in which solubility was determined using a novel application of “Differential Scanning Calorimetry (DSC)”,^26^ resulting in an unusual shape for the α-glycine solubility curve. Second, having further glycine–water solution thermodynamic data (e.g., water activity, enthalpy of dilution and solution heat capacity), for solution compositions in excess of 0.056 mole fraction, would be useful to ensure construction of accurate excess Gibbs free energy models and potentially allow decoupling of uncertainty introduced from the choice of excess Gibbs free energy and eq 16, respectively.

#### Sensitivity Analysis

As shown above, the choice of model used to estimate coefficients in eq 16 impacted solubility predictions at temperatures far from the reference temperature; indicating an underlying model sensitivity. Given that there is limited solubility data in this region (i.e., above 340 K) it is not appropriate to assess reliability of the approach based solely on best fit parameter estimates, as these would be subject to measurement uncertainty. To account for this we perform a sensitivity analysis.

Each aggregated data set (i.e., ln γ_w_, *ΔH*^dil^/*n*_1_, and *c*_p,soln_) used to regress model parameters was used to generate “simulated” data sets. A simulated data set is defined as a data set derived from the original aggregated data set; however, each data point was “blurred” by a random percentage, calculated from *y*_blur_ = *y*_original_ + *y*_original_ϵ where *y* denotes a thermodynamic quantity and ϵ is a random value drawn from a Gaussian distribution with mean μ* = 0, and standard deviation, σ*.

**Figure 6:**
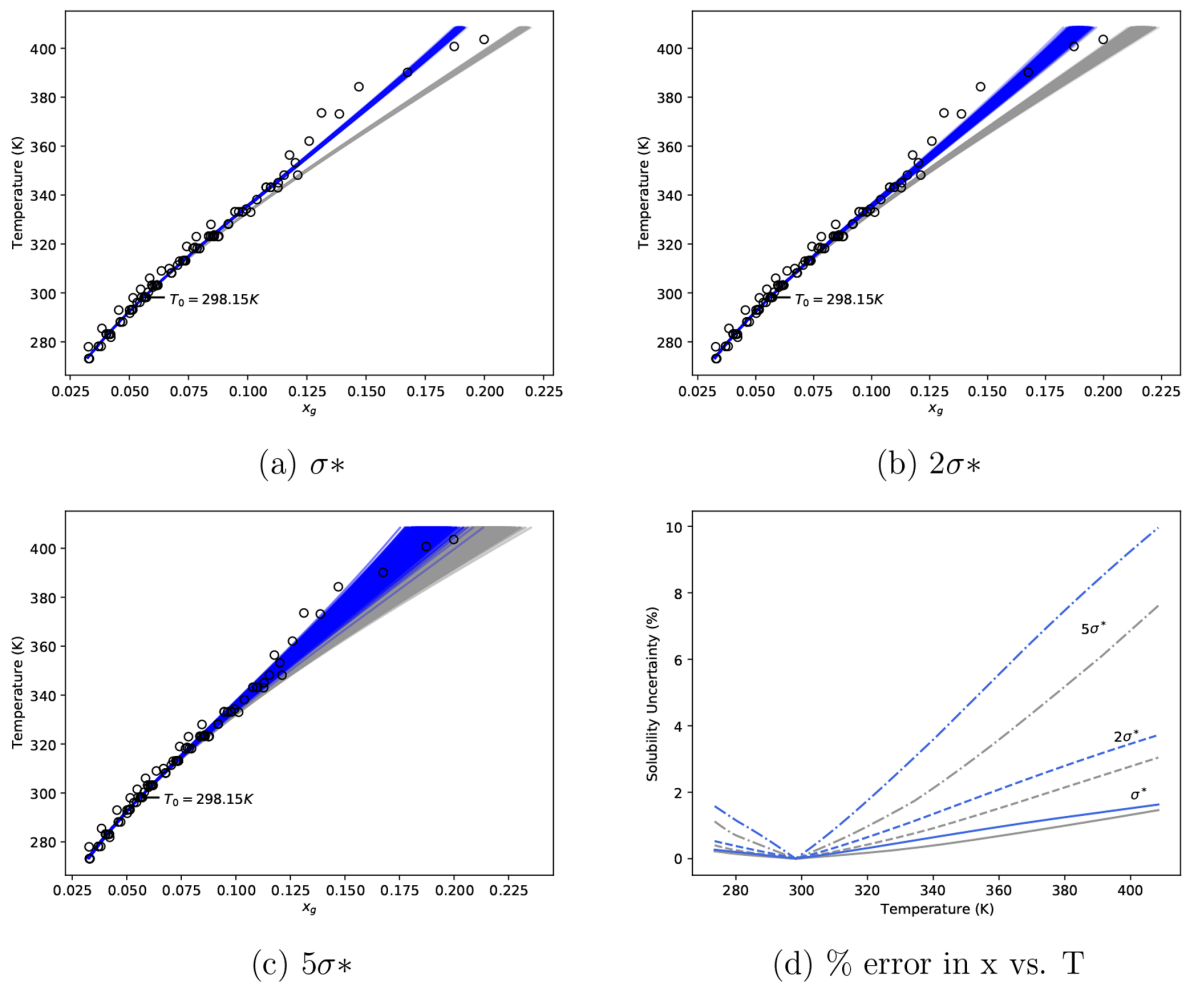
(a–c) Solubility predictions based on excess Gibbs free energy model parameters fit to “simulated” data sets. The gray fan represents the range of predictions for the Scatchard–Hildebrand model, and the blue fan represents the range of predictions of the Scatchard–Hildebrand–Flory–Huggins model. (d) Prediction uncertainty based on fan horizontal width. Gray refers to the Scatchard–Hildebrand model, and blue refers to the Scatchard–Hildebrand–Flory–Huggins model. The open black circles are selected direct measurements labeled as α-glycine^25,26,28−30^ and undefined glycine^9,33−39^

Then, the procedure outlined previously was repeated using simulated data sets, resulting in a new solubility prediction. This process was repeated 1000 times for each excess Gibbs free energy model, giving the predictions presented in Figure 6a. To investigate the effect of uncertainty magnitude, we perform sensitivity analysis at three levels for each thermodynamic quantity; σ*, 2σ*, and 5σ*, where σ* is defined as 0.01, 0.01, and 0.0001 for ln γ_w_, *ΔH*^dil^, and *c*_p,soln_, respectively. It should be noted that the chosen σ* values correspond to approximate uncertainty observed in aggregated data sets shown in Figures 2b, 3a, and 4b. From the available data, measurements of *c*_p,soln_ are more precise, in terms of % error, when compared to other thermodynamic quantities. Uncertainty levels translated to percentage errors are provided in Table 2. Resulting fits to experimental data are presented in the Supporting Information.

**Table 2:** Levels Used in α-Glycine Sensitivity Analysis

	quantity	error (%)
σ*	ln γ_w_	1
	*ΔH*^dil^	1
	*c*_p,soln_	0.01
2σ*	ln γ_w_	2
	*ΔH*^dil^	2
	*c*_p,soln_	0.02
5σ*	ln γ_w_	5
	*ΔH*^dil^	5
	*c*_p,soln_	0.05

From Figure 6, solubility predictions for α-glycine derived from both activity coefficient models are somewhat insensitive to measurement uncertainty (at the levels investigated) between 273–340 K, as illustrated by thin blue and gray bands. However, as the prediction moves further from the reference temperature (298.15 K), prediction uncertainty increases as indicated by a spreading of the bands for all levels. As the level of uncertainty used to “blur” data increases, the uncertainty of predictions far from the reference temperature increases, which is what we expect. It is interesting to note that, even accounting for measurement uncertainty, predictions based on the different excess Gibbs free energy models are significantly different at temperatures far from the reference for predictions based σ* and 2σ* uncertainty levels (Figure 6a and 6b). This suggests that model selection has an impact, independent of measurement uncertainty, on the validity of the method and motivates future work on finding the most appropriate model to collate consistent thermodynamic quantities of liquid solutions. However, at the 5σ* level, we note that prediction bands overlap for the entire temperature range analyzed. From Figure 6d, we note the percentage error for each uncertainty level taken with respect to the best fit solubility predictions for each excess Gibbs free energy model. As shown, the percentage error in solubility increases as the prediction moves from the reference temperature, while at 400 K, the percentage error in solubility prediction is approximately 1, 3, and 8–10% for σ*, 2σ*, and 5σ*, respectively.

**Figure 7:**
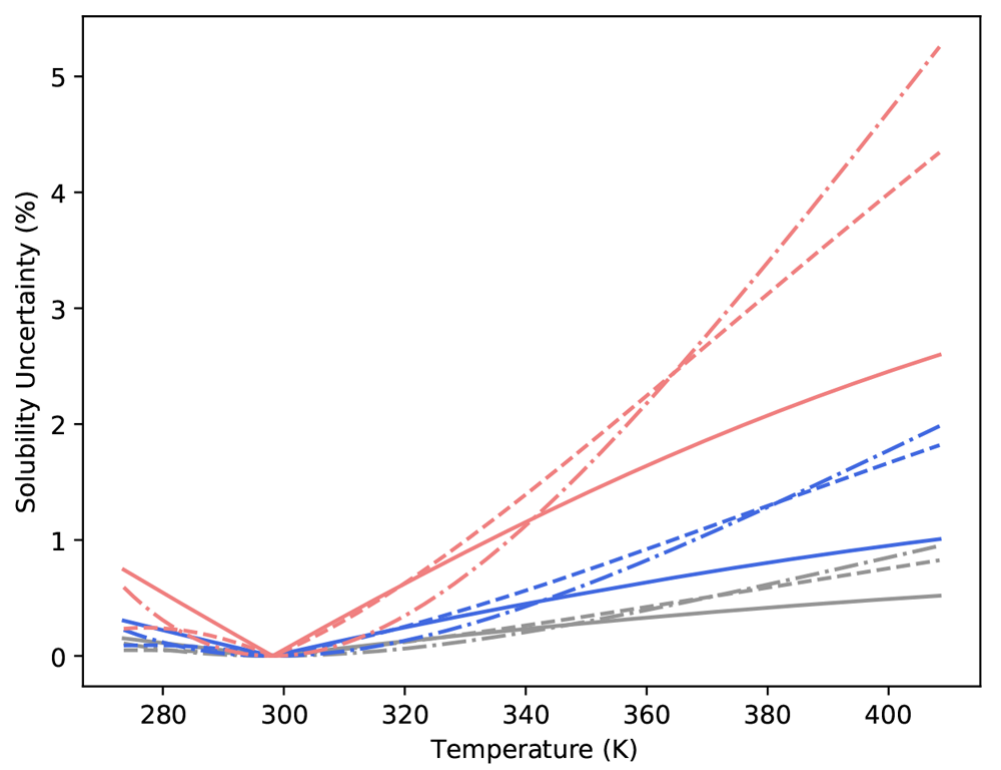
Uncertainty of the predicted α-glycine solubility based on the Scatchard–Hildebrand model with uncertainty of σ* (gray), 2σ* (blue), and 5σ* (red) in the value of ln γ_w_ (solid lines), *ΔH*^dil^/*n* (dashed lines), and *c*_p,soln_ (dashed-dotted lines).

The sensitivity of solubility predictions (based on the Scatchard–Hildebrand excess Gibbs free energy model) to independent thermodynamic data is shown in Figure 7. We note that, for the case where uncertainty in data is estimated by “blurring” data points and generating new model fits, solubility predictions far from the reference temperature are impacted by *c*_p,soln_, *ΔH*^dil^/*n*_g_ and ln γ_w_ in order of most to least impact, where a greater impact corresponds to larger percentage error. If we consider that the level of uncertainty in *c*_p,soln_ is 100 times lower than *ΔH*^dil^ and ln γ_*w*_, it suggests that, for accurate solubility predictions, precise measurements of *c*_p,soln_ are required (i.e., ± 0.1 J mol^–1^ K^–1^). However, it should be noted that, for the same level of uncertainty, both *c*_p,soln_ and *ΔH*^dil^ have a similar impact, while for all levels, uncertainty in ln γ_w_ has comparatively lower impact. For example, at the 5σ* level, the percentage uncertainty in predicted solubility at 400 K is ≈2.5%, 4%, and 5% for ln γ_w_, *ΔH*^dil^, and *c*_p,soln_, respectively.

### β and γ-Glycine Solubility Predictions

In the context of the modeling framework presented for α-glycine, there is scope to predict the solubility of β and γ-glycine, given that direct measurements for *Δh*^∞,°^ and *c*_*p*_^°^ are available (Table 1), alongside regressed excess Gibbs free energy models describing the thermodynamic properties of glycine–water solutions. However, for β-glycine, there is only one (unreliable) report of solubility data available, while for γ-glycine the solubility data is scattered (Figure 1). As discussed, predictions require an estimate for the solubility at the chosen reference temperature around which the expansion is based. While the solubility of α-glycine is accurately known, various data was collated to estimate β and γ-glycine solubility predictions as summarized in Table 3.

**Table 3:** Summary of Data Used to Estimate β- and γ-Glycine Reference Solubility

β	γ
*Δμ*_(298.15 K)_^γ–β^	*Δμ*_(298.15 K)_^α–γ^
(*c*_β_/*c*_α_)_(310 K)_	(*c*_γ_/*c*_α_)_(310 K)_
*T*_E_ and *x*_E_	*T*_E_ and *x*_E_
	*x*_γ_
	*x*_γ_/*x*_α_
	*T*^α–γ^

In Table 3, *Δμ*^*x*–*y*^ is an estimate of the polymorph free energy difference at 298.15 K, (*c*_*x*_/*c*_*y*_)_(310K)_ is polymorph solubility ratios measured in water-antisolvent systems with various solvent compositions at 310 K, *T*_E_ and *x*_E_ are eutectic temperature and composition measurements, *x*_γ_ corresponds to direct solubility measurements of γ-glycine, *x*_α_/*x*_γ_ corresponds to α/γ solubility ratios derived from direct solubility measurements reported together and *T*^α–γ^ corresponds to estimates for the temperature at which the relative stability of α and γ-glycine changes.

#### Eutectic Temperature and Composition

The eutectic temperatures for α-, β-, and γ-glycine in water have been reported by various authors and recently reviewed.^64^ From the available data (see Supporting Information (Eutectic Temperature and Composition)), we note that β-glycine has the most reported measurements, followed by γ and finally α with a single measurement.

**Figure 8:**
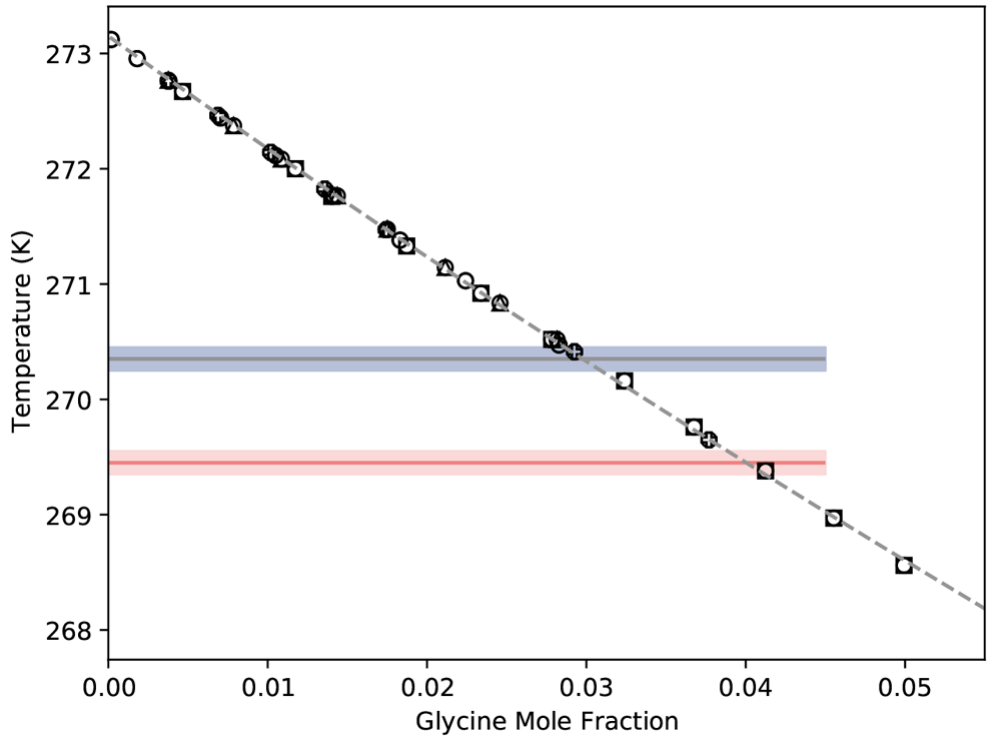
Expected eutectic temperatures and freezing curve The open black symbols are freezing curve measurements,^65−69^ and the gray dashed line is the best fit line. The solid lines are the estimated eutectic temperature, and the opaque bands indicate the uncertainty. Blue refers α-, gray refers to γ-, and red refers to β-glycine.

The eutectic temperatures for β- and γ-glycine are consistent when the reported error is considered, while those reported for α-glycine are conflicting. We assume the reported eutectic temperature of −3.6 °C for α is incorrect on the basis that it is the same as the reported value for β-glycine, and our α-glycine solubility predictions indicate −2.8 °C is a more reasonable value. The expected eutectic temperature for each polymorph was estimated by taking the mean of the reported values and found to be −2.8, −3.7, and −2.8 °C for α, β, and γ, respectively, on which we estimate an uncertainty of ±0.1 °C. The resulting eutectic temperatures are presented alongside selected freezing point measurements in Figure 8.

By evaluating the solution composition at which the eutectic temperature range for β- and γ-glycine intersects the freezing point line, we estimate their eutectic composition; giving *x*_E,β_ = 0.040 ± 0.001 and *x*_E,γ_ = 0.030 ± 0.001 for β and γ, respectively. In the absence of reliable estimates for the solubility of β and γ-glycine at 298.15 K, the eutectic temperature and composition can be used as a point from which we can base our solubility predictions. For example, we can iteratively perform solubility predictions with different reference values and asses the resulting performance in relation to the estimated eutectic temperature and composition. This would work for β-glycine; however, given that the eutectic temperature and composition estimate for γ is the same as α, the utility of the approach is limited for γ-glycine.

#### Bouchard Ratios

Bouchard et al.^6^ report measurements of α-, β-, and γ-glycine solubility in water-antisolvent mixtures of varying solvent composition at 310 K. Measurements for α- and γ-glycine are given in pure water, giving a solubility ratio of *x*_γ_/*x*_α_ = 0.974 ± 0.012. Measurements of β-glycine in pure water are not reported due to rapid recrystallization to α-glycine. However, using available solubility measurement in antisolvent mixtures, we can estimate the solubility ratio of β and α in pure water by extrapolating the solubility ratio from known antisolvent mixtures to pure water (see Supporting Information). We find that this approach gives a reasonable estimate for γ/α, thus apply it to β/γ. The estimated solubility ratio was found to be *x*_β_/*x*_α_ = 1.13 ± 0.08.

#### Polymorph Free Energy Difference Estimates

The free energy differences for glycine polymorphs (α and β) with respect to γ glycine at 298.15 K have been estimated from pure crystal heat measurements at temperatures between 0 and 298.15 K and enthalpy of solution measurements.^22,63,70^ The free energy differences are reported as *Δμ*^α,γ^ = 157 ± 145 J mol^–1^ K^–1^ and *Δμ*^β,γ^ = 277 J mol^–1^ K^–1^, giving *Δμ*^β,α^ = 120 J mol^–1^ K^–1^. It should be noted that the reported uncertainty for *Δμ*^γ,α^ is of the same magnitude as the difference; while no uncertainty was reported for *Δμ*^β,γ^. From thermodynamics, it can be shown that the solubility of two polymorphs at a given temperature is related to the free energy difference:
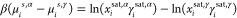
42
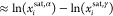
43where we make the assumption that the activity coefficient is approximately the same given that we expect the mole fraction solubility of each polymorph to be reasonably close. From eq 43 we estimate the solubility ratios to be *x*_γ_/*x*_α_ = 0.94 ± 0.05 and *x*_β_/*x*_α_ = 1.05. It should be noted that the mole fraction solubility ratio estimated for γ/α is in good agreement with those derived from direct measurements (Supporting Information).

#### Solubility Predictions

**Figure 9:**
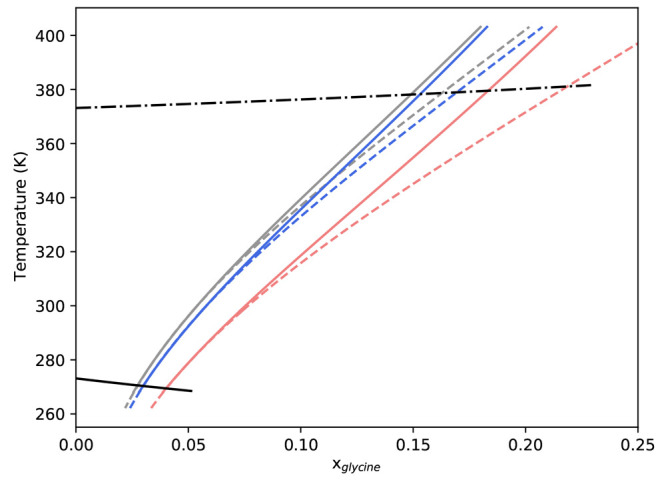
Solubility predictions for α-, β-, and γ-glycine based on eq 16, with coefficients derived from different glycine–water solution models. The dashed lines are the prediction of the Scatchard–Hildebrand model, and the solid lines are the predictions of the Scatchard–Hildebrand–Flory–Huggins model. Blue refers to α-, red refers to β-, and gray refers to γ-glycine solubility in water. The black solid line is the best fit to the freezing point depression curve, and the black dash-dotted line is the ideal boiling curve.

**Figure 10:**
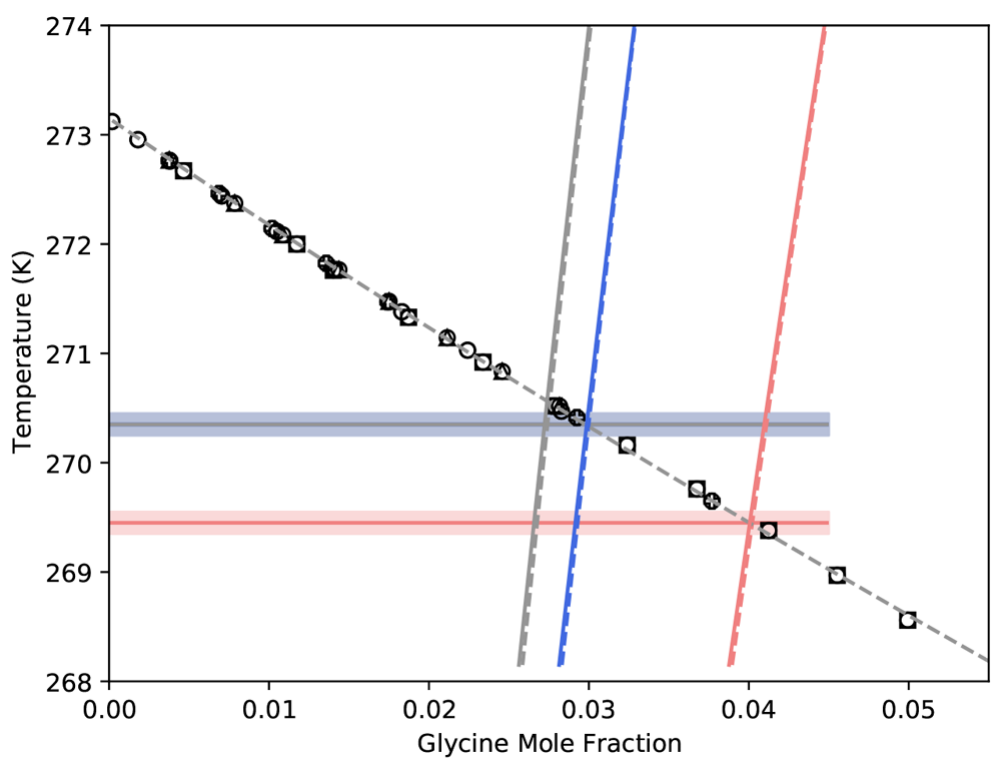
Eutectic temperature and composition predictions α-, β-, and γ-glycine based on eq 16, with coefficients derived from different glycine–water solution models. Open circles are freezing point measurements for water,^65−69^ and the gray dashed line is the best-fit line. Blue refers to α-, red refers to β-, and gray refers to γ-glycine. The dashed lines are the predictions of the Scatchard–Hildebrand, and the solid lines are the predictions of the Scatchard–Hildebrand–Flory–Huggins model.

**Figure 11:**
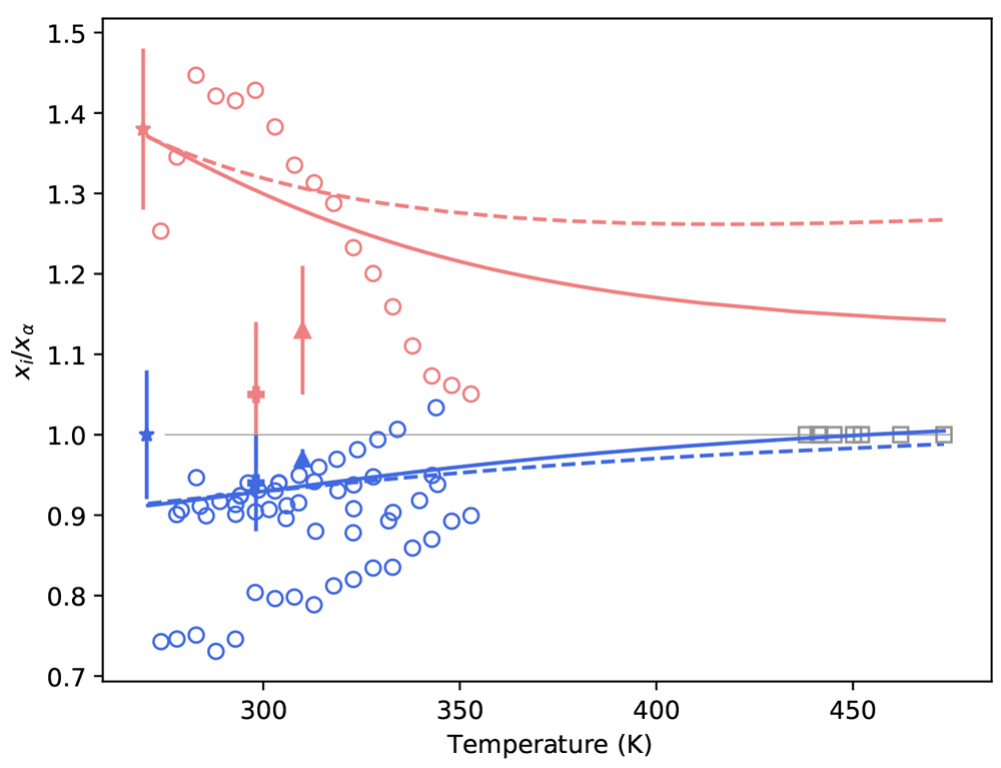
Glycine polymorph mole fraction solubility ratio predictions based on eq 16, with coefficients derived from different glycine–water solution models. Blue refers to the γ–α ratio, and red refers to β–α ratio. The dashed lines is the prediction of the Scatchard–Hildebrand model, and the solid lines Scatchard–Hildebrand–Flory–Huggins model. The open symbols are ratios derived from direct measurements^65−69^ (see Supporting Information), and the pluses are estimates from free energy ratios,^63^ stars are estimates from eutectic temperatures and freezing point data, triangles are estimates from ref (6), and gray squares are α–γ crossover estimates.^22,26,71−76^ Bars indicate uncertainty estimates.

On the basis of the discussion above, solubility predictions for β and γ-glycine were developed based on the following. For β-glycine, the solubility prediction was fixed on the expected eutectic temperature of −3.7 °C using an iterative approach to find the “best” reference solubility at *T*_0_ = 298.15 K, which was found to be 0.074 and 0.073 for the Scatchard–Hildebrand and Scatchard–Hildebrand–Flory–Huggins solution models, respectively. For γ-glycine, the ratio estimated by the free energy difference was used, giving *x*_γ,298.15_ = 0.052 (based on *x*_α,298.15_ = 0.056). The resulting solubility, solubility ratio, and eutectic point estimates are presented in Figures 9–11, respectively.

As expected, the choice of the excess Gibbs free energy model has a similar impact on solubility predictions for β and γ-glycine, as was seen for α-glycine, resulting in diverging solubility predictions away from the reference temperature. As shown in Figure 9, the effect of excess Gibbs free energy model on solubility predictions for γ-glycine is similar to α-glycine—the predictions begin to diverge around 340 K. However, it is interesting to note predictions for β-glycine diverge much closer to the reference temperature (i.e., 298.15 K). This is likely a result of β-glycine having a much higher solubility at 298.15 K, which, as shown in Figure 2, corresponds to diverging estimates for ∂ ln γ_w_/∂*x*_*g*_ and ∂^2^ ln γ_w_/∂*x*_g_^2^, for each excess Gibbs free energy model. As such, we suggest 273.15–310 K as reliable range for our β-glycine solubility predictions. However, we expect reliability could be improved with accurate water activity data solution compositions above 0.055 glycine mole fraction. This further highlights that prediction accuracy of the presented approach can be improved through more accurate estimation of partial derivatives of relevant thermodynamic quantities.

In addition, the mole fraction solubility ratios (across a wide range of temperature) are similarly impacted. It is interesting to note (as shown in Figure 11) the Scatchard–Hildebrand–Flory–Huggins model correctly predicts the γ/α crossover in the range of temperatures reported in literature; however, the Scatchard–Hildebrand model fails to do so, despite coming very close. This could be interpreted as an indication that the composition dependence of the water activity coefficient is better described by the Scatchard–Hildebrand–Flory–Huggins model. Both solution models predict β-glycine to be least stable (i.e., highest solubility) in the temperature range explored, agreeing with qualitative observations in the literature. However, predictions for β/α based on the expected eutectic temperature appear to be inconsistent with independent ratio estimates derived from antisolvent mixture solubility data and free energy difference estimates, though this could be attributed to significant uncertainty associated with both estimates.

Finally, we note that solubility predictions based on different solution models are approximately identical in the eutectic region (as shown in Figure 10), where the predicted eutectic temperature and composition for α-glycine agrees extremely well with data available in literature, as does γ (noting that models for α and γ were not based on the eutectic temperature at all). Our predictions suggests that −2.7 °C is an appropriate eutectic temperature for γ-glycine.

## Conclusions

A novel approach to estimate the temperature dependence of solubility was presented that relies on experimentally accessible thermodynamic data at a single temperature. The approach was applied the glycine–water system, and it was found that, between 273–340 K, the approach provided a solubility prediction that agreed very well with available direct solubility measurements for α-glycine.

Sensitivity analysis was used to assess accuracy of solubility predictions with respect to underlying measurement uncertainty, as well as underlying excess free energy models used to derive required thermodynamic quantities. This provided a range of plausible solubility predictions far from the chosen reference temperature, and in particular above 340 K, where there is lack of reliable solubility data for glycine. Finally, we applied the approach to β and γ-glycine where previous solubility data is inconsistent or limited, providing estimates for their aqueous solubility between 273–310 and 273–330 K, respectively.

The approach introduced here provides a novel framework for how various thermodynamic data can be used in concert to predict the temperature dependent solubility of crystal polymorphs. This will be useful for systems where direct measurements of solubility are challenging for one or more polymorphs, and compounds that undergo thermal decomposition or polymorph transition prior to melting.

## Supporting Information Available

The following files are available free of charge. The Supporting Information is available free of charge at https://pubs.acs.org/doi/10.1021/acs.cgd.1c01217.

Solubility data fits, freezing point data fits, model parameter definitions, supplementary sensitivity analysis figures, additional derivations, and tabulated solubility predictions (PDF)
