# Supplementary material for: Temperature Dependence of Solubility Predicted from Thermodynamic Data Measured at a Single Temperature: Application to α, β, and γ-Glycine
Source: Cryst Growth Des. 2022 Feb 8;22(3):1691–706. doi: 10.1021/acs.cgd.1c01217 (PMC9008547; doi:10.1021/acs.cgd.1c01217)
Supplement: Supplementary file 1 — cg1c01217_si_001.pdf [file cg1c01217_si_001.pdf]

Temperature Dependence of Solubility Predicted from Thermodynamic Data Measured at a Single Temperature: Application to α, β and γ-Glycine - Supporting Information

Andrew Manson,† Jan Sefcik,†,‡ and Leo Lue*,†

†*Department of Chemical and Process Engineering, University of Strathclyde, James Weir Building, 75 Montrose Street, Glasgow G1 1XJ, UK*

‡ *EPSRC Continuous Manufacturing & Advanced Crystallisation (CMAC) Future Manufacturing Research Hub, University of Strathclyde, Glasgow G1 1RD, UK*

E-mail: leo.lue@strath.ac.uk

Derivation of Differential Solubility Equation (Based on Williamson (1944)

Here we present a complete derivation from fundamental thermodyamic arguments for Eq.(1) in the associated manuscript. We attribute this derivation to the work of Williamson (1944)[1] and present it here as a resource for interested readers.

The Gibbs free energy change for a hypothetical thermodynamic reaction (at equilibrium) is defined as:

$$\Delta G = G^{products} - G^{reactants} \tag{1}$$

$$= \Delta H - T\Delta S, \tag{2}$$

where ΔH and ΔS are the enthalpy and entropy change associated with the reaction, respectively, and T is temperature. The Gibbs-Helmholtz equation is given as:

$$\frac{d}{dT}\left(\frac{\Delta G}{T}\right) = -\frac{\Delta H}{T^2}, \tag{3}$$

which relates the temperature dependence of the Gibbs free energy of the hypothetical process to the enthalpy change of the process. As an aside, we can interpret ΔG further. For the reversible reaction $\mathrm{solute}(s) \leftrightarrow \mathrm{solute}(aq)$, which describes the equilibrium between a pure solid solute and a saturated solution:

$$\Delta G = G^{products} - G^{reactants} \tag{4}$$

$$= n_1\mu_1^{l} - n_1\mu_1^{s,\circ} \tag{5}$$

$$\Delta g = \left(\mu_1^{l,\circ} + RT\ln a_1\right) - \mu_1^{s,\circ}, \tag{6}$$

where 1 denotes solute, $\mu_1^{s,\circ}$ is the molar Gibbs free energy of the pure solid solute, $\mu_1^{l,\circ}$ is the molar Gibbs free energy of the solute in solution at the chosen reference state, and μ_1^{l} is the

partial molar Gibbs free energy of the solute (i.e. chemical potential) in solution. For a chemical reaction at equilibrium, $\Delta G = 0$, which means $\Delta g = 0$, thus:

$$\Delta g_1^\circ = -RT \ln a_1 \tag{7}$$

where a_1 is the solute activity, and we introduce $\Delta g_1^\circ = \mu_1^{l,\circ} - \mu_1^{s,\circ}$ as the standard molar Gibbs free energy for the dissolution process (i.e. the difference between the partial molar Gibbs free energy between the pure solid solute and the solute in solution, in their standard states, which is typically interpretted in terms of the melting properties of the solute). Alternatively, as described by Williamson, the standard Gibbs free energy change for the dissolution equilibrium reaction can be expressed in terms of the equilibrium constant K via:

$$\Delta g_1^\circ = -RT \ln K, \tag{8}$$

where $K = a_1$. Following[1] and combining either Eq. (7) or Eq. (8) with Eq. (3) we note that:

$$\frac{d \ln a_1}{dT} = \frac{(h_1^\infty - h_1^{\circ,s})}{RT^2}, \tag{9}$$

where $h_1^\infty - h_1^{l,\circ}$ is defined as the standard enthalpy change for the dissolution process (i.e. $\mathrm{solute}(s) \leftrightarrow \mathrm{solute}(aq)$), which is also referred to as the experimentally measurable enthalpy of solution to infinite dilution (noting that we use the infinitely dilute reference state to define a). Further, we note that $a = \gamma x$. Now, we need to define the derivative on the left hand side. Noting that the relationship is only valid along the solubility curve:

$$\left(\frac{d \ln (x_1 \gamma_1)}{dT}\right)_{sat} = \left(\frac{d \ln x_1}{dT}\right)_{sat} + \left(\frac{d \ln \gamma_1}{dT}\right)_{sat}. \tag{10}$$

As described by,[1] special attention should be given to $\left(\frac{d \ln \gamma_1}{dT}\right)_{sat}$, because it is both a function of temperature and composition, both of which vary as we move along the solubility curve. We

can do so by introducing the total derivative:

$$d\ln\gamma_1 = \left(\frac{\partial \ln\gamma_1}{\partial T}\right)_{\ln x} dT + \left(\frac{d\ln\gamma_1}{\partial \ln x_1}\right)_T d\ln x_1, \tag{11}$$

which gives (imposing the constraint that differentiation occurs along the solubility curve):

$$\left(\frac{d\ln\gamma_1}{dT}\right)_{sat} = \left(\frac{\partial \ln\gamma_1}{\partial T}\right)_{\ln x} + \left(\frac{d\ln\gamma_1}{\partial \ln x}\right)_T \left(\frac{d\ln x_1}{dT}\right)_{sat}. \tag{12}$$

Here, we note that:

$$\left(\frac{\partial \ln\gamma_1}{\partial T}\right)_{\ln x} = -\frac{(\bar{h}_1 - h_1^{\infty})}{RT^2}, \tag{13}$$

which is often termed the 'relative partial molar heat content of the solute'. Coupled with the standard enthalpy change for the dissolution process:

$$\Delta h_1^{soln,s} = \frac{(\bar{h}_1 - h_1^{\infty})}{RT^2} + \frac{(h_1^{\infty} - h_1^{s,\circ})}{RT^2} \tag{14}$$

$$= \frac{(\bar{h}_1 - h_1^{s})}{RT^2}. \tag{15}$$

Combining all of the above, we get:

$$\frac{d\ln x_1}{dT} + \left(\frac{\partial \ln\gamma_1}{\partial \ln x_1}\right)_T \left(\frac{d\ln x_1}{dT}\right)_{sat} = \frac{(\bar{h}_1 - h_1^{s})}{RT^2} \tag{16}$$

$$\frac{d\ln x_1}{dT} = \frac{1}{RT^2}\left[\frac{(\bar{h}_1 - h_1^{s})}{1 + \partial \ln\gamma_1/\partial \ln x_1}\right]. \tag{17}$$

Solubility Model Coefficient Definitions and Values

Definitions for model coefficients and their interpretation are presented in Table 1, nomenclature is defined in Table 2 and coefficients estimated for both free energy models and all polymorphs are presented in Table 3.

Table 1: Taylor series expansion coefficient definitions

Coefficient	Definition
a_0	$(\bar{h}_1^{\,l}(T_0, x_{1,\text{sat}}(T_0)) - h_1^{s,\circ}(T_0))$
a_1	$T_0(\bar{c}_{p,1}^{l}(T_0, x_{1,\text{sat}}(T_0)) - c_{p,1}^{s,\circ}(T_0))$
a_2	$x_{1,\text{sat}}(T_0)(\partial(\bar{h}_1^{\,l}(T_0, x_{1,\text{sat}}(T_0)) - h_1^{s,\circ}(T_0))/\partial x_1)$
b_0	$(x_{2,\text{sat}}(T_0)\partial \ln \gamma_2(T_0, x_{1,\text{sat}}(T_0))/\partial x_1)$
b_1	$-\beta_0 \left[x_{1,\text{sat}}(T_0)(\partial(\bar{h}_1^{\,l}(T_0, x_{1,\text{sat}}(T_0)) - h_1^{s,\circ}(T_0))/\partial x_1)\right]$
b_2	$x_{1,\text{sat}}(T_0)(-\partial \ln \gamma_2(T_0, x_{1,\text{sat}}(T_0))/\partial x_1 + x_{2,\text{sat}}(T_0)\partial^2 \ln \gamma_2(x_{1,\text{sat}}(T_0))/\partial x_1^2)$

Table 2: Thermodynamic property definitions

Property	Name	Definition
$\bar{h}_1^l$	Solute partial molar enthalpy (solution)	$\bar{h}_1^l = h_1^\infty + \frac{\partial \ln \gamma_1^*}{\partial \beta}$
$h_1^{s,\circ}$	Pure solute molar enthalpy (solid)	$h_1^{s,\circ} = h_1^\infty + \Delta h^{s,\infty}$
$\bar{c}_{p,1}^l$	Solute partial molar heat capacity (solution)	$\bar{c}_{p,1}^l = c_{p,1}^{l,\circ} + \frac{\partial^2 \ln \gamma_1}{\partial T \partial \beta}$
$c_{p,1}^{s,\circ}$	Pure solute molar heat capacity (solid)	
$\ln \gamma_i$	Activity coefficient for component i (Raoult's Law)	
$\ln \gamma_i^*$	Activity coefficient for component i (Henry's Law)	$\ln \gamma_i^* = \frac{\ln \gamma_i}{\ln \gamma_i^\infty}$
$\ln \gamma_i^\infty$	Activity coefficient at infinite dilution	$\lim_{x \to 0} \ln \gamma_i$

Table 3: Taylor series expansion coefficient values (α, β and γ-glycine)

Coefficient	Scatchard-Hildebrand	Exp. Scatchard-Hildebrand
	α	
a_0	13103.5	13103.5
a_1	-96.8	-96.8
a_2	-671.1	-671.1
b_0	0.093	0.07
b_1	0.27	0.27
b_2	-0.045	-0.15
	β	
a_0	12597.	12605.
a_1	1314.5	1261.0
a_2	-622.2	-625.4
b_0	0.079	0.019
b_1	0.251	0.252
b_2	-0.056	-0.240
	γ	
a_0	13421.5	13421.5
a_1	478.8	478.8
a_2	-677.8	-677.8
b_0	0.096	0.081
b_1	0.273	0.273
b_2	-0.041	-0.131

Solubility Fits for Estimating Solubility Ratio

Certain solubility data reports contain measurements for both α and γ-glycine. In theory, these can be processed to determine the solubility ratio of polymorphs at various temperatures. In some cases, measurements are for both polymorphs are reported at the same temperature; however, in others, they are reported at different temperatures.

For data sets reporting data at different temperatures for each polymorph, the following solubility model was used to interpolate a uniform set of solubility, thus giving estimates for the solubility ratio:

$$\ln x = \frac{-a}{R}\frac{1}{T} + \frac{b}{R}\ln T + c \tag{18}$$

Table 4: α and γ-glycine parameters for Eq. (18) regression

	Park et. al		Yang et. al		Igarashi et. al	
Parameter	α	γ	α	γ	α	γ
$a(\times 10^3)$	1.213	6.401	4.435	2.858	1.141	0.144
b	1.041	24.908	−8.911	−3.773	0.371	4.220
c	−4.738	−166.390	62.676	25.756	−1.236	−26.583

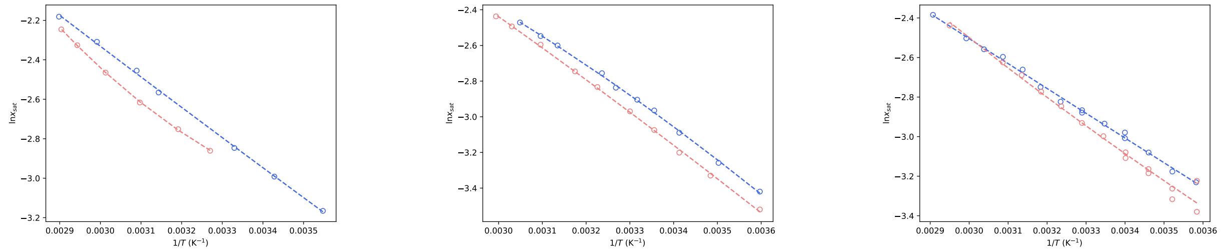

(a) Park et. al.

(b) Yang et. al.

(c) Igarashi et. al.

Figure 1: Fits to selected literature solubility data (blue; α, red; γ, open circles; data, dashed lines; fits)

Bouchard Solubility Ratio Data and Interpretation

Bouchard et al., measured the solubility of α, β and γ-glycine in aqueous solutions of varying composition containing methanol, ethanol, 2-propanol or acetone at 310 K. β-glycine solubility in pure water wasn't measured due to assumed rapid re-crystallisation to α. As shown in Fig. 2, extrapolating the solubility ratio (c_γ/c_α) measured in various mixed solvent systems (above 70 wt% water) gives a reasonable estimate for the solubility ratio in pure water. Thus, the solubility ratio (c_β/c_α in pure water was estimated using data from mixed solvent solubility measurements - shown graphically in Fig. 2.

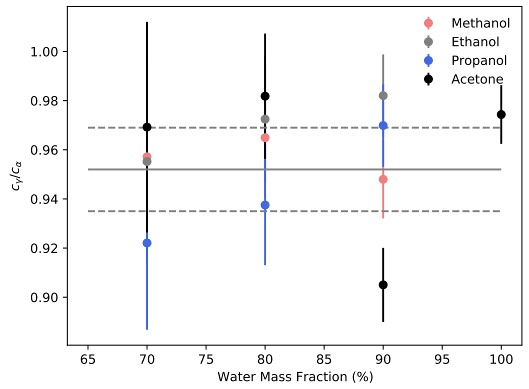

(a) γ-α

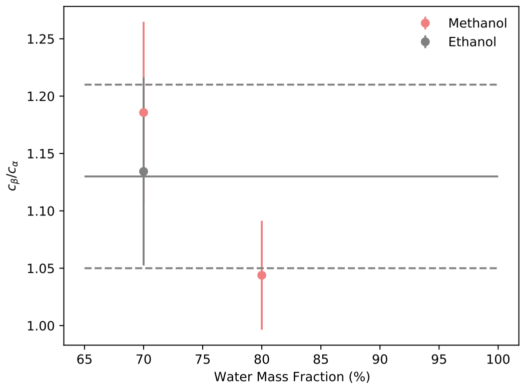

(b) β-α

Figure 2: Selected Bouchard solubility ratios with estimated uncertainties (points; solubility ratios (note: c measured in g glycine / g solution, grey solid line; expected solubility ratio, grey dashed lines; $\pm\sigma^*$)

The uncertainty on ratio estimates derived from Bouchard anti-solvent solubility data was estimated using Uncertainties python package. For γ-α, the solubility ratio uncertainty estimate was calculated using uncertainty data provided in the article (i.e. $\pm$ 0.002 g/gsoln) and translated into mole fraction ratios. For the purpose of estimation, we apply the same uncertainty to the 'average' solubility ratio for β-α.

Miscellaneous Tabulated Literature Data

In this section, literature data used in various figures throughout the associated manuscript are tabulated for a complete record. This includes: α-γ cross-over temperatures and α, β and γ-glycine eutectic temperatures.

Table 5: Literaure α-γ cross-over temperatures

Reference	Temperature (K)
[2]	452.14
[3]	441.55–473.15
[4]	438.15 ± 5
[5]	450.15
[6] (via [7])	438
[8]	462.15
[9]	441.15 & 445.15
[7]	425–450

Table 6: Literaure eutectic temperature and composition estimates (adapted from a review by[10])

Reference	α	β	γ
[10]	-2.8 ± 0.1	-3.6 ± 0.1	-2.8 ± 0.1
[11]	-3.6	-3.8	-2.9
[12]		-3.6	-2.7
[13]		-3.6 ± 0.1	

Freezing Point Depression

In general, the presence of solute in solvent leads to 'freezing point depression' (i.e. a decrease in the temperature at which a solvent freezes). It should be noted that freezing point depression occurs while the concentration of solute in solution is below the eutectic composition for the solute/solvent system. The freezing point of glycine-water solutions (or related properties from which the freezing point can be derived) has been measured by various authors[14–18] and presented in Fig. 3. It should be noted that reports of water osmotic coefficients at the freezing point were converted to freezing point temperatures using:

$$\Delta T \approx \frac{\Phi}{1.858} m_g,$$

where $\Delta T = 0°C - T_{fp}$. In addition, the freezing point depression was predicted using:

$$\ln x_w = \frac{\Delta H^{fus}}{R} \left(\frac{1}{T} - \frac{1}{T_m} \right),$$

where $\Delta H^{fus} = 6010$ J/mol and $T_m = 273.15$ K (i.e. the melting properties of pure ice). From Fig. 3, four of the data sets are consistent, while the data reported by Shimoyamada et. al., appears to be inconsistent. The consistency of the other four data sets is further supported by modeling work of Rowland.[19] The following model was used to correlate the consistent data:

$$\ln x_w = -A \left(\frac{1}{T} - \frac{1}{T_m} \right) + B \ln \left(\frac{T}{T_m} \right),$$

where $A = 10115.62$ K and $B = -34.29$, and is shown in Fig. 3.

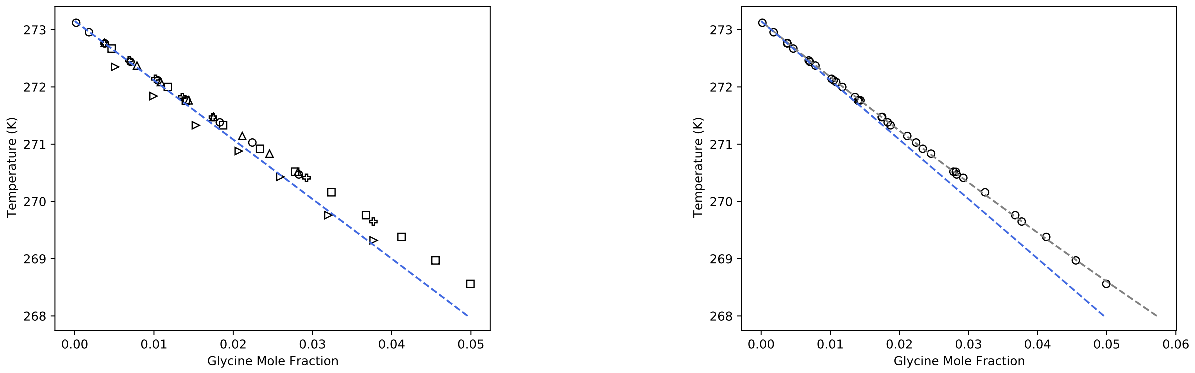

(a) All data

(b) Selected data with best fit

Figure 3: Freezing point depression data (symbols; experimental data, blue dahsed line; prediction from ice melting properties, grey dashed line; best fit)

Sensitivity Analysis

In the associated manuscript, we present a range of predictions for the aqueous solubility of α-glycine, based on a sensitivity analysis of the underlying thermodynamic data used to regress excess Gibbs free energy models, as well as sensitivity to the form of the chosen models. Here we illustrate the aforementioned sensitivity, for all levels of assumed measurement uncertainty for $\ln \gamma_w$, $\Delta H^{dil}/n_g$ and c_p^{soln}. Figures are grouped together based on level of assumed measurement uncertainty (i.e. σ^*, $2\sigma^*$ and $5\sigma^*$) - as described in the associated manuscript. Finally, we present α-glycine aqueous solubility predictions based on underlying measurement uncertainty to each themrodynamic quantity (i.e. $\ln \gamma_w$, ΔH^{dil} and c_p^{soln}) at each uncertainty level, for the Scatchard-Hildebrand model.

Uncertainty Level: σ^*

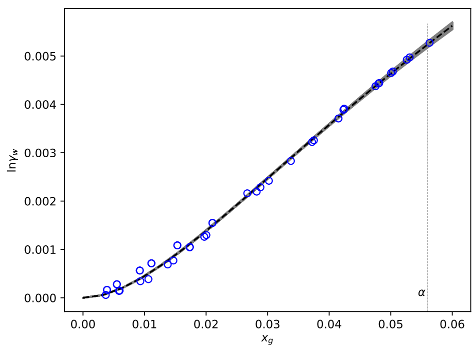

(a) Scatchard-Hildebrand

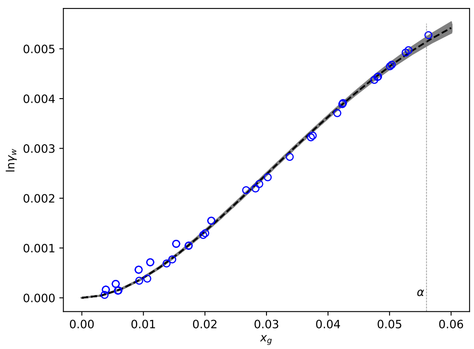

(b) Scatchard-Hildebrand-Flory-Huggins

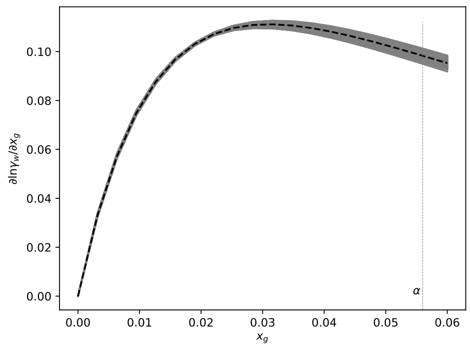

(c) Scatchard-Hildebrand

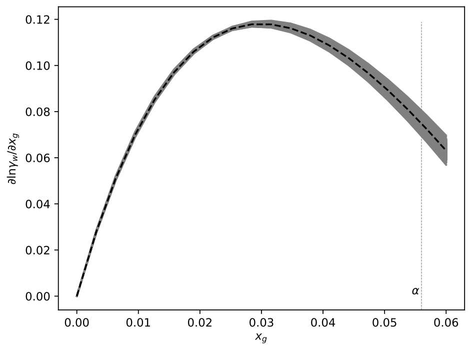

(d) Scatchard-Hildebrand-Flory-Huggins

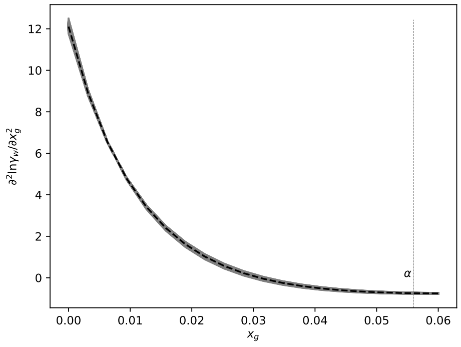

(e) Scatchard-Hildebrand

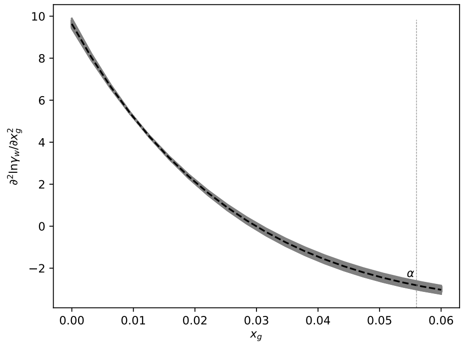

(f) Scatchard-Hildebrand-Flory-Huggins

Figure 4: Sensitivity of water activity and associated partial derivatives in glycine-water mixtures at 298.15 K to assumed measurement uncertainty of σ^*. (a)–(b) show $\ln \gamma_w$ (where blue open circles are selected data from literature as discussed in the associated manuscript), (b)–(c) show $\partial \ln \gamma_w / \partial x_g$ and (d)–(e) show $\partial^2 \ln \gamma_w / \partial x_g^2$. Vertical dashed line indicates α-glycine aqueous solubility at 298.15 K

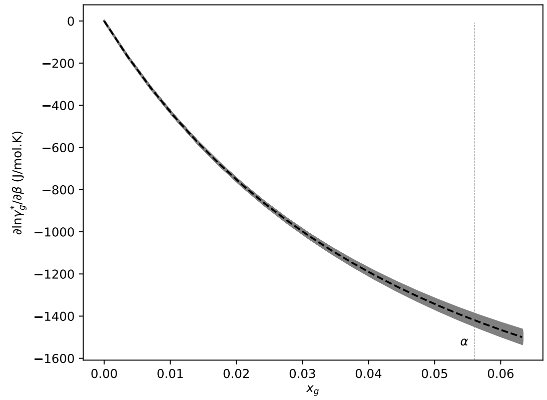

(a) $\partial \ln \gamma_w^* / \partial \beta$

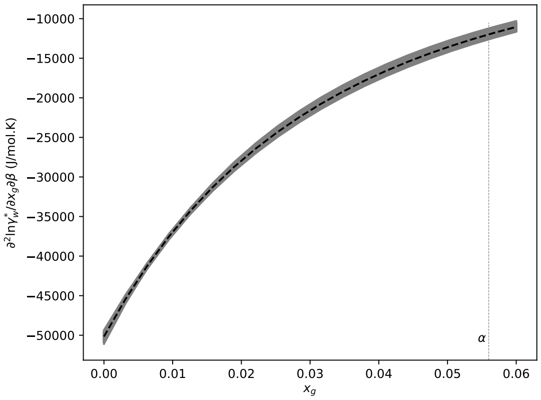

(b) $\partial^2 \ln \gamma_w^* / \partial x_g \partial \beta$

Figure 5: Sensitivity of $\partial \ln \gamma_g^* / \partial \beta$ and $\partial^2 \ln \gamma_g / \partial x_g \partial \beta$ at 298.15 K to assumed measurement uncertainty in $\Delta H^{dil} / n_g$. Vertical dashed line indicates α-glycine aqueous solubility at 298.15 K

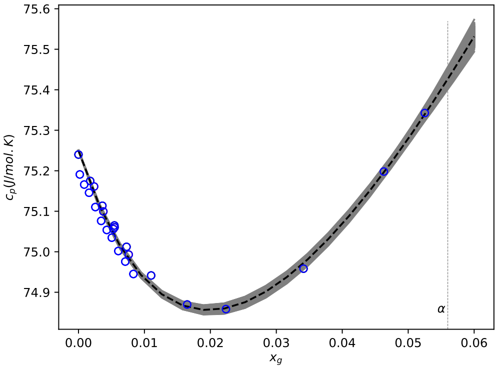

(a) c_p

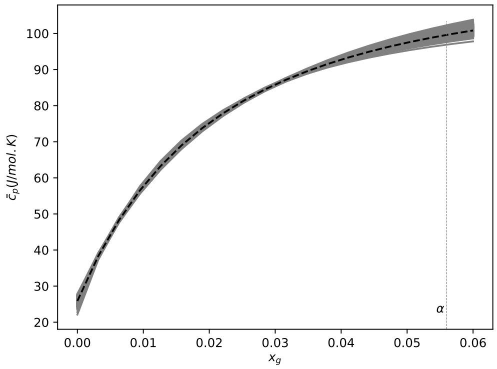

(b) $\bar{c}_{p,g}$

Figure 6: Sensitivity of glycine-water solution heat capacity and glycine partial molar heat capacity in glycine-water mixtures at 298.15 K to assumed measurement uncertainty in c_p^{soln}. Blue open circles indicate selected literature data. Vertical dashed line indicates α-glycine aqueous solubility at 298.15 K

Uncertainty Level: 2 σ^*

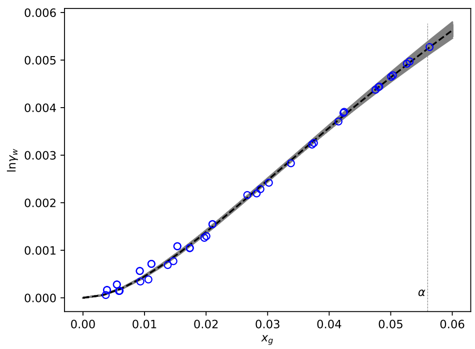

(a) Scatchard-Hildebrand

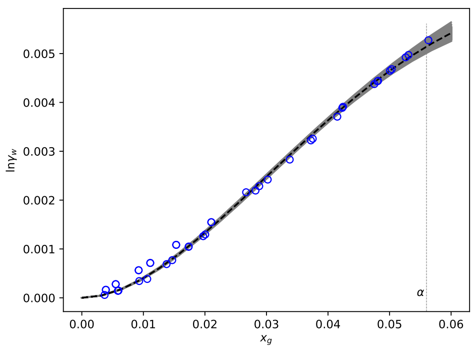

(b) Scatchard-Hildebrand-Flory-Huggins

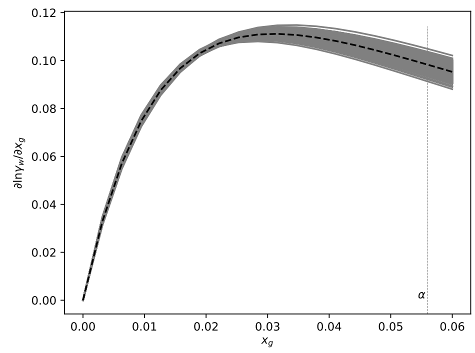

(c) Scatchard-Hildebrand

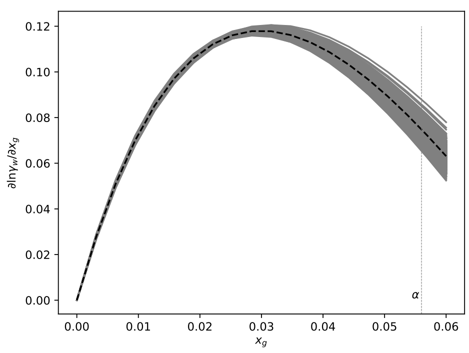

(d) Scatchard-Hildebrand-Flory-Huggins

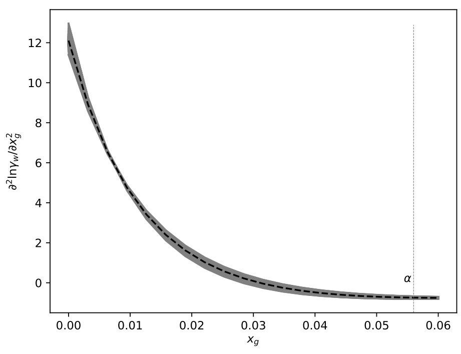

(e) Scatchard-Hildebrand

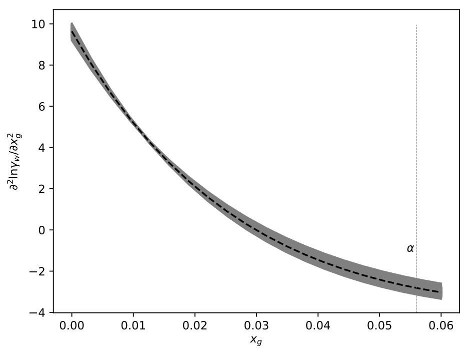

(f) Scatchard-Hildebrand-Flory-Huggins

Figure 7: Sensitivity of water activity and associated partial derivatives in glycine-water mixtures at 298.15 K to assumed measurement uncertainty of $2\sigma^*$. (a)–(b) show $\ln\gamma_w$ (where blue open circles are selected data from literature as discussed in the associated manuscript), (b)–(c) show $\partial\ln\gamma_w/\partial x_g$ and (d)–(e) show $\partial^2\ln\gamma_w/\partial x_g^2$. Vertical dashed line indicates α-glycine aqueous solubility at 298.15 K

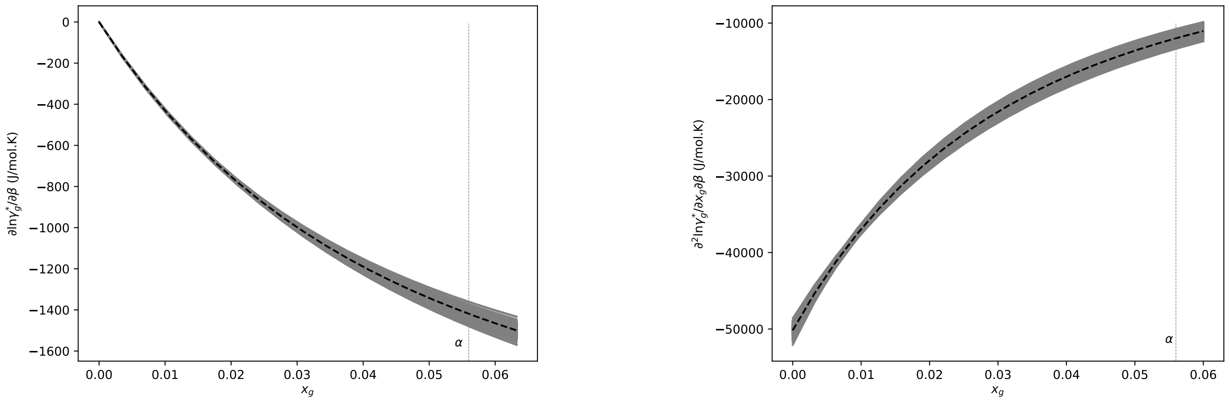

(a) $\partial \ln \gamma_w^* / \partial \beta$

(b) $\partial^2 \ln \gamma_w^* / \partial x_g \partial \beta$

Figure 8: Sensitivity of $\partial \ln \gamma_g^* / \partial \beta$ and $\partial^2 \ln \gamma_g / \partial x_g \partial \beta$ at 298.15 K to assumed measurement uncertainty in $\Delta H^{dil} / n_g$. Vertical dashed line indicates α-glycine aqueous solubility at 298.15 K

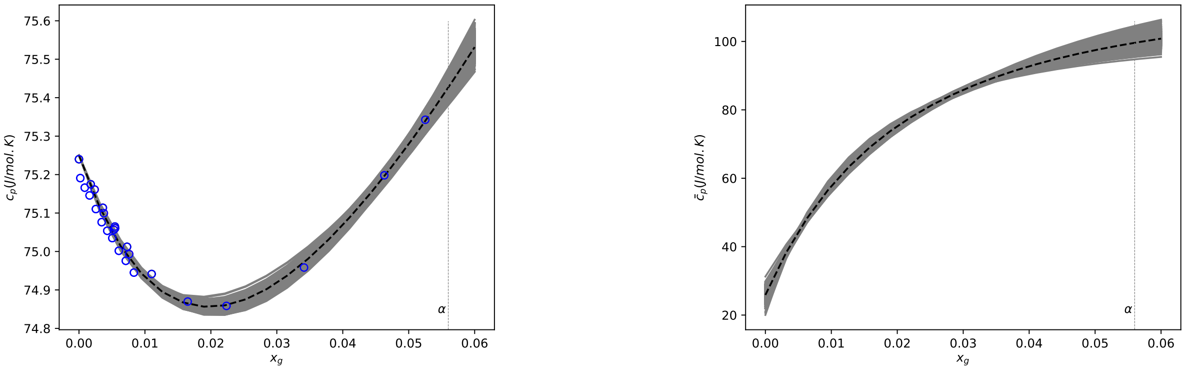

(a) c_p

(b) $\bar{c}_{p,g}$

Figure 9: Sensitivity of glycine-water solution heat capacity and glycine partial molar heat capacity in glycine-water mixtures at 298.15 K to assumed measurement uncertainty in c_p^{soln}. Blue open circles indicate selected literature data. Vertical dashed line indicates α-glycine aqueous solubility at 298.15 K

Uncertainty Level: 5 σ^*

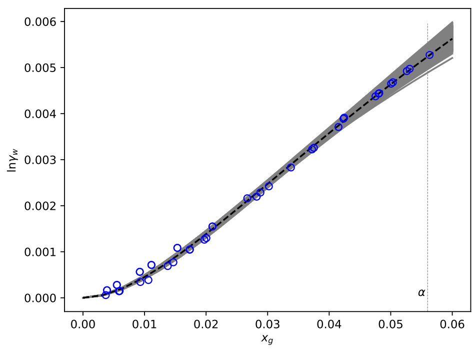

(a) Scatchard-Hildebrand

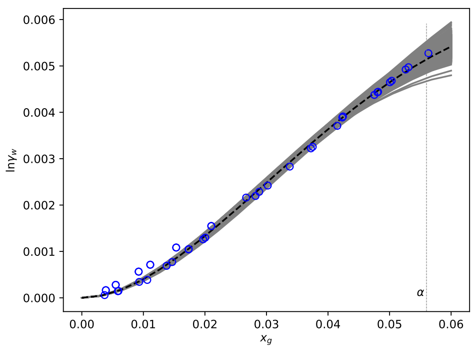

(b) Scatchard-Hildebrand-Flory-Huggins

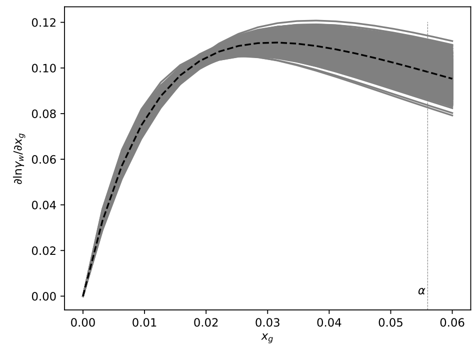

(c) Scatchard-Hildebrand

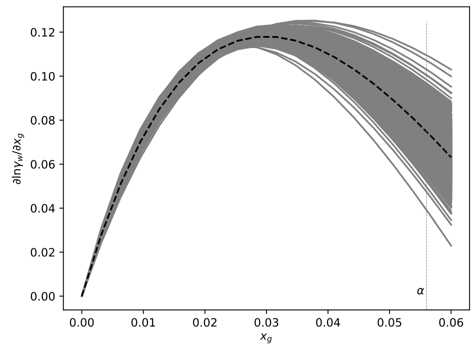

(d) Scatchard-Hildebrand-Flory-Huggins

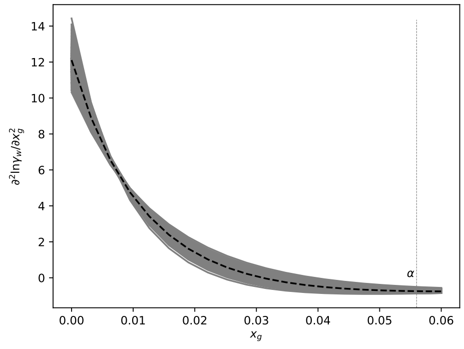

(e) Scatchard-Hildebrand

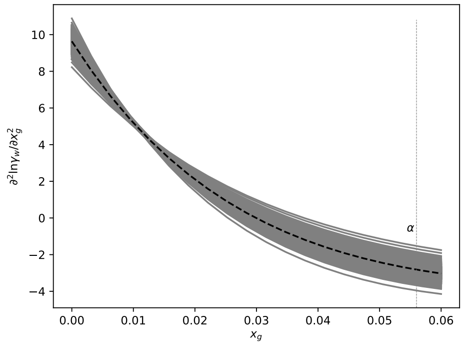

(f) Scatchard-Hildebrand-Flory-Huggins

Figure 10: Sensitivity of water activity and associated partial derivatives in glycine-water mixtures at 298.15 K to assumed measurement uncertainty of $5\sigma^*$. (a)–(b) show $\ln \gamma_w$ (where blue open circles are selected data from literature as discussed in the associated manuscript), (b)–(c) show $\partial \ln \gamma_w / \partial x_g$ and (d)–(e) show $\partial^2 \ln \gamma_w / \partial x_g^2$. Vertical dashed line indicates α-glycine aqueous solubility at 298.15 K

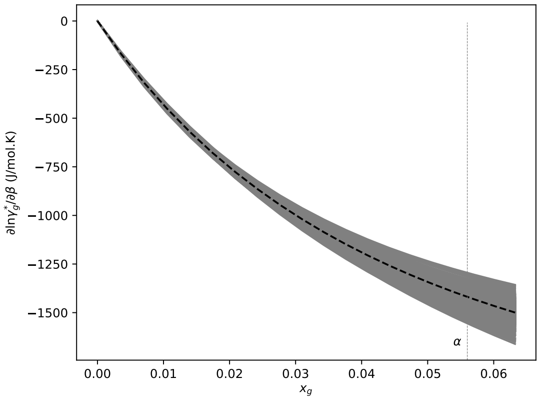

(a) $\partial \ln \gamma_w^* / \partial \beta$

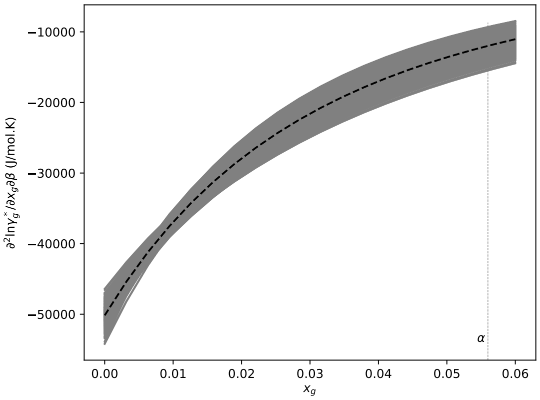

(b) $\partial^2 \ln \gamma_w^* / \partial x_g \partial \beta$

Figure 11: Sensitivity of $\partial \ln \gamma_g^* / \partial \beta$ and $\partial^2 \ln \gamma_g / \partial x_g \partial \beta$ at 298.15 K to assumed measurement uncertainty in $\Delta H^{dil} / n_g$. Vertical dashed line indicates α-glycine aqueous solubility at 298.15 K

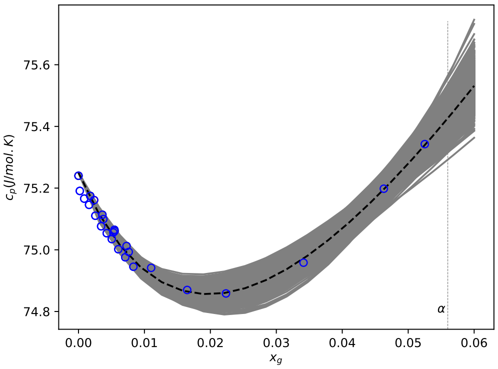

(a) c_p

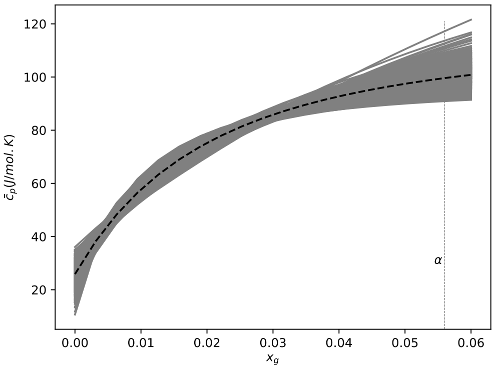

(b) $\bar{c}_{p,g}$

Figure 12: Sensitivity of glycine-water solution heat capacity and glycine partial molar heat capacity in glycine-water mixtures at 298.15 K to assumed measurement uncertainty in c_p^{soln}. Blue open circles indicate selected literature data. Vertical dashed line indicates α-glycine aqueous solubility at 298.15 K

α-Glycine Solubility Predictions

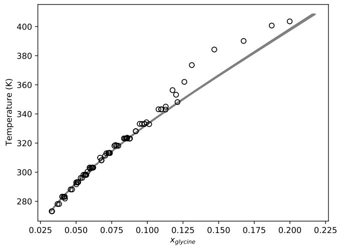

(a) $\ln \gamma_w (1\%)$

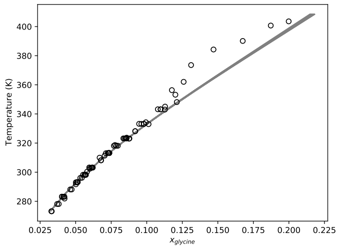

(b) $\Delta H^{dil}/n_g(1\%)$

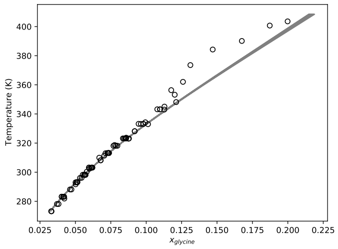

(c) $c_{p,soln}(0.01\%)$

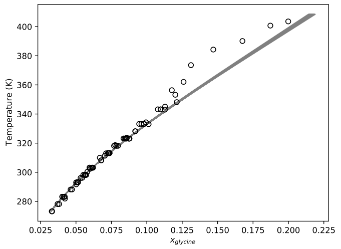

(d) $\ln \gamma_w (2\%)$

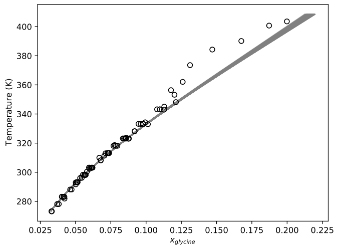

(e) $\Delta H^{dil}/n_g(2\%)$

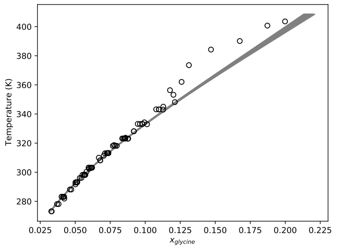

(f) $c_{p,soln}(0.02\%)$

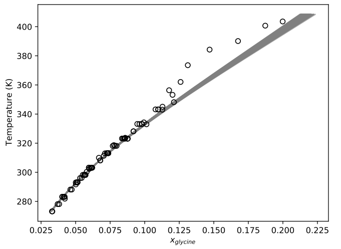

(g) $\ln \gamma_w (5\%)$

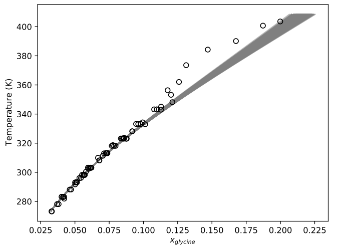

(h) $\Delta H^{dil}/n_g(5\%)$

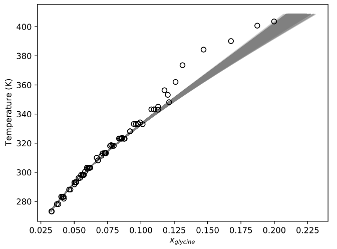

(i) $c_{p,soln}(0.05\%)$

Figure 13: α-glycine solubility prediction sensitivity to independent sources of thermodynamic data using the Scatchard-Hildebrand model (black circles; direct solubility measurements, grey fan; solubility predictions)

Tabulated Solubility Predictions

In the associated manuscript, we graphically present various predictions for the aqueous solubility of α, β and γ-glycine across a broad range of temperatures. To make re-use of this data simpler, here we present tabulated values corresponding to each prediction at 5 K increments between 263.15 and 398.15 K, where the solubility is given in terms of glycine mole fraction.

Table 7: Solubility predictions for α, β and γ-glycine in water at 5 K increments between 263.15 and 398.15 K derived from predictions described in the associated manuscript. Solubility reported in glycine mole fraction. SH and SHFH denote predictions based on Scatchard-Hildebrand and Scatchard-Hildebrand-Flory-Huggins excess Gibbs free energy models, respectively.

	α		β		γ	
T (K)	SH	SHFH	SH	SHFH	SH	SHFH
263.15	0.025	0.024	0.034	0.034	0.023	0.022
268.15	0.028	0.028	0.039	0.039	0.026	0.026
273.15	0.032	0.032	0.044	0.044	0.029	0.029
278.15	0.036	0.036	0.049	0.049	0.033	0.033
283.15	0.041	0.041	0.055	0.055	0.038	0.038
288.15	0.046	0.046	0.061	0.061	0.042	0.042
293.15	0.051	0.051	0.067	0.067	0.047	0.047
298.15	0.056	0.056	0.074	0.073	0.052	0.052
303.15	0.062	0.061	0.081	0.079	0.057	0.057
308.15	0.067	0.067	0.088	0.086	0.063	0.063
313.15	0.074	0.073	0.096	0.093	0.069	0.068
318.15	0.080	0.079	0.104	0.099	0.075	0.074
323.15	0.086	0.085	0.112	0.106	0.081	0.080
328.15	0.093	0.091	0.120	0.113	0.088	0.086
333.15	0.100	0.097	0.129	0.120	0.095	0.092
338.15	0.107	0.103	0.138	0.127	0.102	0.098
343.15	0.115	0.109	0.146	0.134	0.109	0.105
348.15	0.122	0.116	0.156	0.141	0.116	0.111
353.15	0.129	0.122	0.165	0.148	0.123	0.117
358.15	0.137	0.128	0.174	0.155	0.131	0.124
363.15	0.145	0.134	0.184	0.161	0.139	0.130
368.15	0.152	0.141	0.193	0.168	0.146	0.136
373.15	0.160	0.147	0.203	0.175	0.154	0.143
378.15	0.168	0.153	0.213	0.181	0.162	0.149
383.15	0.176	0.159	0.223	0.188	0.170	0.155
388.15	0.184	0.165	0.232	0.195	0.178	0.162
393.15	0.192	0.171	0.242	0.201	0.186	0.168
398.15	0.200	0.177	0.252	0.207	0.194	0.174

References

(1) Williamson, A. T. The Exact Calculation of Heats of Solution From Solubility Data. *Trans. Faraday Soc.* **1944**, *40*, 421.

(2) Srinivasan, K. Crystal Growth of α and γ Glycine Polymorphs and Their Polymorphic Phase Transformations. *J. Cryst. Growth* **2008**, *311*, 156–162.

(3) Perlovich, G. L.; Hansen, L. K.; Bauer-Brandl, A. The polymorphism of glycine: Thermochemical and structural aspects. *J. Therm. Anal. Calorim.* **2001**, *66*, 699–715.

(4) Iitaka, Y. The crystal structure of γ-glycine. *Acta Crystallogr.* **1961**, *14*, 1–10.

(5) Park, K.; Evans, J. M. B.; Myerson, A. S. Determination of Solubility of Polymorphs Using Differential Scanning Calorimetry. *Cryst. Growth Des.* **2003**, *3*, 991–995.

(6) Kozhin, V. M. Thermal expansion tensors of α-, β-, and γ-modifications of glycine. *Kristallografiya* **1978**, *23*, 1211–15.

(7) Boldyreva, E. V.; Drebushchak, V. A.; Drebushchak, T. N.; Paukov, I. E.; Kovalevskaya, Y. A.; Shutova, E. S. Polymorphism of glycine, Part II. *J. Therm. Anal. Calorim.* **2003**, *73*, 419–428.

(8) Balakrishnan, T.; Babu, R. R.; Ramamurthi, K. Growth, Structural, Optical and Thermal Properties of γ-glycine Crystal. *Spectrochim. Acta, Part A* **2008**, *69*, 1114–1118.

(9) Bhat, M.; Dharmaprakash, S. Growth of Nonlinear Optical γ-glycine Crystals. *J. Cryst. Growth* **2002**, *236*, 376–380.

(10) Drebushchak, V. A.; Ogienko, A. G.; Boldyreva, E. V. Polymorphic Effects At the Eutectic Melting in the H2O-Glycine System. *J. Therm. Anal. Calorim.* **2012**, *111*, 2187–2194.

(11) Yu, L.; Huang, J.; Jones, K. J. Measuring Free-Energy Difference Between Crystal Polymorphs Through Eutectic Melting. *J. Phys. Chem. B* **2005**, *109*, 19915–19922.

(12) Chongprasert, S.; Knopp, S. A.; Nail, S. L. Characterization of Frozen Solutions of Glycine. *J. Pharm. Sci.* **2001**, *90*, 1720–1728.

(13) Shalaev, E.; Malakhov, D.; Kanev, A.; Kosyakov, V.; Tuzikov, F.; Varaksin, N.; Vavilin, V. Study of the Phase Diagram Water Fraction of the System Water-Glycine-Sucrose By DTA and X-Ray Diffraction Methods. *Thermochim. Acta* **1992**, *196*, 213–220.

(14) Anslow, G. A.; Foster, M. L.; Klinger, C. The Absorption Spectra of Glycine Solutions and Their Interpretation. *J. Biol. Chem.* **1933**, *103*, 81–92.

(15) Lewis, W. C. M. The Crystallization, Denaturation and Flocculation of Proteins With Special Reference To Albumin and Hemoglobin; Together With an Appendix on the Physicochemical Behavior of Glycine. *Chem. Rev.* **1931**, *8*, 81–165.

(16) Huang, J.; Stringfellow, T. C.; Yu, L. Glycine Exists Mainly As Monomers, Not Dimers, in Supersaturated Aqueous Solutions: Implications for Understanding Its Crystallization and Polymorphism. *J. Am. Chem. Soc.* **2008**, *130*, 13973–13980.

(17) Shimoyamada, M.; Shibata, M.; Ishikawa, K. I.; Fukuta, Y.; Ishikawa, S.; Watanabe, K. Freezing and Eutectic Points of an Aqueous Amino Acid Solution Containing Ethanol, and the Effect of Ethanol Addition on the Freeze Concentration Process. *Biosci Biotechnol Biochem* **1994**, *58*, 836–838.

(18) Scatchard, G.; Prentiss, S. S. Freezing Points of Aqueous Solutions. VII. Ethyl Alcohol, Glycine and Their Mixtures. *J. Am. Chem. Soc.* **1934**, *56*, 1486–1492.

(19) Rowland, D. Thermodynamic Properties of the Glycine + H2O System. *J. Phys. Chem. Ref. Data* **2018**, *47*, 023104.
